# Higher-order temporal network effects through triplet evolution

**DOI:** 10.1038/s41598-021-94389-w

**Published:** 2021-07-29

**Authors:** Qing Yao, Bingsheng Chen, Tim S. Evans, Kim Christensen

**Affiliations:** 1 Blackett Laboratory and Centre for Complexity Science, Imperial College London, South Kensington Campus, London, SW7 2AZ UK; 2 School of Systems Science, Beijing Normal University, Beijing, 100875 China

**Keywords:** Applied mathematics, Statistical physics, thermodynamics and nonlinear dynamics

## Abstract

We study the evolution of networks through ‘triplets’—three-node graphlets. We develop a method to compute a transition matrix to describe the evolution of triplets in temporal networks. To identify the importance of higher-order interactions in the evolution of networks, we compare both artificial and real-world data to a model based on pairwise interactions only. The significant differences between the computed matrix and the calculated matrix from the fitted parameters demonstrate that non-pairwise interactions exist for various real-world systems in space and time, such as our data sets. Furthermore, this also reveals that different patterns of higher-order interaction are involved in different real-world situations. To test our approach, we then use these transition matrices as the basis of a link prediction algorithm. We investigate our algorithm’s performance on four temporal networks, comparing our approach against ten other link prediction methods. Our results show that higher-order interactions in both space and time play a crucial role in the evolution of networks as we find our method, along with two other methods based on non-local interactions, give the best overall performance. The results also confirm the concept that the higher-order interaction patterns, i.e., triplet dynamics, can help us understand and predict the evolution of different real-world systems.

## Introduction

The collective behaviour of a complex system cannot be understood and predicted by considering the basic units of the system in isolation^1,2^. Complex networks^3,4^ are often used to represent complex systems and thenin which the basic unit of interaction, the edge, represents pairwise interactions. However, in systems represented by networks there are often local processes, known as higher-order interactions, where groups of more than two participants interact and the set of pairwise interactions does not capture the whole picture^5^. Research has revealed that many empirical systems display higher-order interactions, for example social systems^6–8^, neuroscience^9–11^, biology^12^, and ecology^13^. Therefore to understand the dynamics of real complex systems we often need to describe interactions beyond the simplest pairwise relationships.

Hypergraphs^14–16^ naturally encode higher-order interactions in terms of fundamental units, their hyperedges. However, in many cases the data only reveals pairwise interactions making the simple graph the appropriate representation and the higher order interactions have to be inferred from the pairwise interactions recorded in a network. For instance, data on phone calls does not reveal other higher-order connections, e.g. through face-to-face meetings, but the pattern of calls can reveal the existence of such connections. Network Science offers many approaches to discovering these local group interactions from a network. It is natural to work with small sub-graphs, motifs^6,17^, or if we consider induced subgraphs (the maximal subgraph defined by a given vertex set), in terms of graphlets^18,19^. Higher order analysis in terms of paths is important in several contexts^20,21^ but more relevant here is the use of cliques, subgraphs which are complete graphs. Cliques have long played an important role in social science, for instance see^22^, and can be used in many contexts such as community detection^23,24^. Cliques in networks are the basis for analysis in terms of simplicial complexes as used in algebraic topology. In network analysis, simplicial complexes^25–27^ have been used to analyse network geometry^28^, to model structure in temporal networks^29^, investigate synchronization phenomenon^30–33^, social contagion^34^, epidemic spreading^35^, and neuroscience^10,11^.

One area where network analysis is less well developed is the temporal evolution of networks. Many complex systems are not static so an important application of networks is to analyse their behaviour over time^36–43^. Using higher-order interactions in an evolving network context has been considered in a few contexts^34,44–46^. Most of the research on motifs in temporal networks focuses on how the number of each type of motif changes as the network is evolves. On the other hand, the study of simplicial complexes is interested in how the fully connected triangles affect the structures of networks. So there is a gap between understanding how three-node combinations will evolve and how that evolution will affect the evolution of the whole network. This gap motivates us to design a method to investigate whether any non-pairwise interactions will be observed by measuring three-node dynamics.

**Figure 1:**
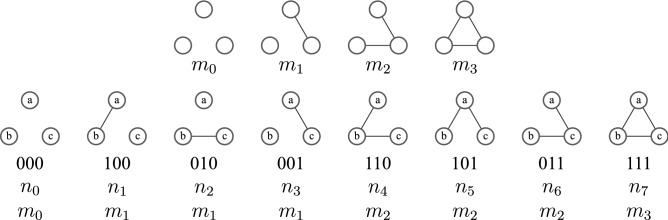
In the top row are the four triplet states of ${\mathscr {M}}_4$, used only for illustration in this paper. The four states $m_{i} \in {\mathscr {M}}_4$ ($i = 0,1,2,3$) are characterised by the number of links *i* in the given three-node graph. In the lower row, there are the eight distinct states of triplets when nodes are distinct (labelled). Below each labelled diagram is the binary representation of the edge set, the label $n_i \in {\mathscr {M}}_8$ ($i = 0,1\dots 7$), and the corresponding $m_i$ triads if labels are ignored.

In this paper we study higher-order processes in temporal networks through the simplest examples of a higher-order interactions, the evolution of the node triplet, three nodes and all three potential edges between these three nodes. Our method looks at the evolution of a network through the relationships of three nodes as shown in the top row of Fig. 1. so the higher-order structures include a clique, the triangle, and a path of length two. Studying the evolution of relationships in a network by focussing on just three nodes has a long history as this includes the process where a path of length two connecting three nodes turns into a triangle as in triadic closure^7,8,47,48^ which underpins many other concepts such as structural holes^49^.

This paper is organised in the following way: In the first section, we introduce the transition matrix that describes the Markovian evolution of the triplet. In the second section, we propose a null model that can demonstrate the higher-order interactions indeed exist in many real-world networks. In the third part, based on the triplet evolution, we create an algorithm that can predict the existence of the links in the temporal networks.

## Triplet dynamics

In order to look beyond pairwise interactions we focus on triplets, at the configurations of three nodes in a network along with all their edges, i.e. the three-node graphlet (also known as a “triphlet”). configurations of three nodes in a network along with any edges between those nodes as shown in Fig. 1. Our focus is not on the number of each triplet type at each time but on how each triplets evolves from one arrangement to the next in a temporal network, an example of “temporal graphlets”.

### Transition matrix

We start with temporal graphs ${\mathscr {G}}(s)$, a sequence of graphs with one node set ${\mathscr {V}}$ but with variable edges sets ${\mathscr {E}}(s)$, where *s* is a discrete time variable. The variable $\mathsf {M}_s(u,v,w)$, often abbreviated to $\mathsf {M}_s$, records the state of a three-node triplet (*u*, *v*, *w*) at time *s*. The states are the subgraphs equivalent to the three nodes and all the edges between them at time *s*. That is $\mathsf {M}_s\equiv \mathsf {M}_s(u,v,w)$ is a map from a node triplet (*u*, *v*, *w*), where $u,v,w \in {\mathscr {V}}$, to the induced graphlet, the maximal subgraph in ${\mathscr {G}}(s)$ containing the nodes *u*, *v* and *w*. We will use ${\mathscr {M}}$ to denote the set of all the possible three-node graphs and $m_{i} \in {\mathscr {M}}$ represents one of the possible graphs in ${\mathscr {M}}$. So formally

1 $$\begin{aligned} \begin{array}{rccl} \mathsf {M}_{s}:&{} {\mathscr {V}}\times {\mathscr {V}}\times {\mathscr {V}}&{}\rightarrow &{} {\mathscr {M}},\\ &{} (u,v,w) &{}\mapsto &{} \mathsf {M}_{s}(u,v,w) = m_i . \end{array} \end{aligned}$$

The choice of ${\mathscr {M}}$ is not unique and it depends on the characteristics of the nodes and links used to distinguish configurations. If we characterise the states by the number of links among the three nodes, that is use unlabelled graphs, there are just four distinct states in ${\mathscr {M}}$ which we can name as $m_0$, $m_1$, $m_2$ and $m_3$ as shown in  Fig. 1. So ${\mathscr {M}}_4$ is the state set characterised by the number of links and here $m_i$ is the unlabelled graph of three nodes and *i* edges. We will use ${\mathscr {M}}_4$ for visualisation and illustration only in our work here.

By contrast, we want to consider the labels (identities) of the nodes in our work. So for our results we work with eight states in ${\mathscr {M}}$ since each link between the three pairs of nodes can either be present or absent. That means the links between different pairs of nodes are distinct. This state set is represented by ${\mathscr {M}}_{8}$ as illustrated in the lower row of Fig. 1.

Typically one uses graphlets by counting the frequency of each graphlet $m \in {\mathscr {M}}$ in each graph ${\mathscr {G}}(s)$ in the temporal graph sequence. So a simple way to look at the evolution is to see how these counts change. We wish to go beyond this and look at the way local structure controls the local evolution. In order to do this we define a transition matrix $\mathsf {T}$ which describes the likelihood that a given triplet is to be transformed in to another triplet in one time step. That is $\mathsf {T}_{ij}(s)$ gives the probability that a triplet of nodes in the state $m_i$ in ${\mathscr {G}}(s-1)$ at time $s-1$ becomes the triplet $m_j$ in ${\mathscr {G}}(s)$ at the next time step, *s*. That is

2 $$\begin{aligned} \mathsf {T}_{ij}(s) = {\mathrm {Pr}}(\mathsf {M}_{s} = m_j| \mathsf {M}_{s-1} = m_i) \, . \end{aligned}$$

By definition, all entries of $\mathsf {T}$ are non-negative, $\mathsf {T}_{ij} (s) \ge 0$, and each row in the transition matrix satisfies a normalisation condition

3 $$\begin{aligned} \sum _{j} \mathsf {T}_{ij} (s) = \sum _{m_j \in {\mathscr {M}}}{\mathrm {Pr}}(\mathsf {M}_{s} = m_j| \mathsf {M}_{s-1}=m_i) =1 \text {,} ~ \forall \; m_i \in {\mathscr {M}}\, . \end{aligned}$$

In practice we have to use an estimate $\widehat{\mathsf {T}}(s)$ for the transition matrix $\mathsf {T}(s)$. We do this by using a subset ${\mathscr {T}}_{s-1}$ of all possible distinct node triplets so ${\mathscr {T}}_{s-1} \subseteq {\mathscr {V}}^3$. From this subset of node triplets, we then count how often the associated graphlet transforms from $m_i$ to $m_j$. More formally we define

4a $$\begin{aligned} \widehat{\mathsf {T}}_{ij} (s)= & {} \frac{1}{k_i} \sum _{(u,v,w) \in {\mathscr {T}}_{s-1}} \delta (\mathsf {M}_{s}(u,v,w),m_j) \; \delta (\mathsf {M}_{s-1}(u,v,w),m_i) \, , \end{aligned}$$

4b $$\begin{aligned} k_i= & {} \sum _j \sum _{(u,v,w) \in {\mathscr {T}}_{s-1}} \delta (\mathsf {M}_{s}(u,v,w),m_j) \; \delta (\mathsf {M}_{s-1}(u,v,w),m_i) \end{aligned}$$

where $\delta ({\mathscr G}_1,{\mathscr G}_2)=1$ (0) if graphlets ${\mathscr G}_1$ and ${\mathscr G}_2$ are isomorphic (not isomorphic). The best estimate $\widehat{\mathsf {T}}_{ij} (s)$ of $\mathsf {T}_{ij}(s)$ is produced if ${\mathscr T}_{s-1}$ is the set of all possible distinct node triplets. However, for a large graph with *N* nodes there are $$\left( {\begin{array}{c}N\\ 3\end{array}}\right) = N\cdot (N-1)\cdot (N-2)/6$$ three node combinations making it computationally inefficient to use all triplets. For example, for $$N = 100,000, \left( {\begin{array}{c}N\\ 3\end{array}}\right) = 1.7\cdot 10^{14}$$, Therefore, we will use random sampling of triplets of a sufficient amount to produce our estimates. The estimations are detailed in Appendix A.

**Figure 2:**
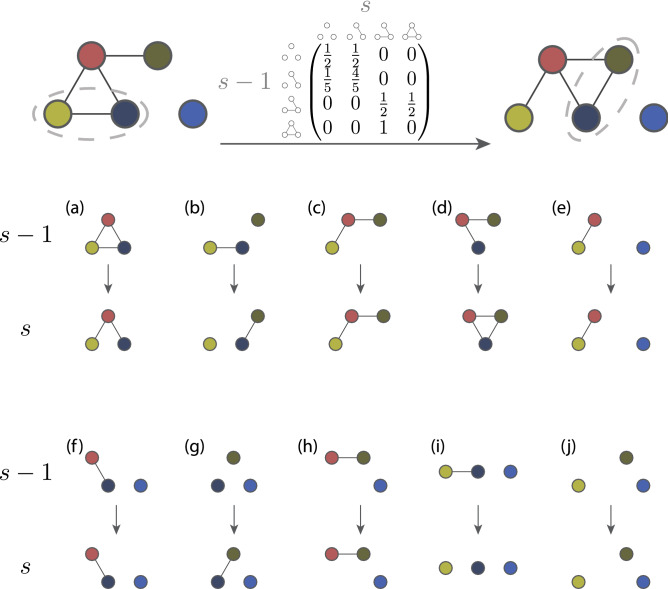
An illustration of how the empirical transition matrix $\widehat{\mathsf {T}}(s)$, shown top centre, can be computed from evolution of the network ${\mathscr G}(s-1)$ shown top left, with links at time $s-1$, to ${\mathscr G}(s)$ shown top right, with links at time *s*. When the states are only characterised by the number of links of the three-node combinations, ${\mathscr M}_4$, for illustrative purposes, there are four possible states being denoted by $m_i$ ($i = 0, 1, 2, 3$) of Fig. 1. The subscript *i* represents the number of the links *i* in the graphlet $m_i$, so $m_0$ is the triplet with zero links, $m_{1}$ is a triplet with one link and so on. There are $$\left( {\begin{array}{c}5\\ 3\end{array}}\right) = 10$$ ways of choosing 3 nodes for a set of 5 nodes, and the evolution of all ten triplets are shown in the remaining rows in the figure above. For instance, the subgraph induced by a triplet (a) is the triplet $m_3$. This triplet loses an link in the next graphlet, so the same node triplet is now associated with the two link triplet $m_2$. This change, therefore, contributes to the $\widehat{\mathsf {T}}_{32}$ entry. As this is the only induced subgraph (graphlet) in the earlier ${\mathscr G}(s-1)$ graph which is isomorphic to the $m_3$ triad, this means $\widehat{\mathsf {T}}_{32}=1$ while $\widehat{\mathsf {T}}_{3j}=0$ for $j = 0,1,3$. On the other hand, the double link $m_2$ triad appears twice as an induced subgraph of node triplets in ${\mathscr G}(s-1)$, namely (c) and (d). These triplets have two different triads in ${\mathscr G}(s)$ leading to two non zero entries in the $\widehat{\mathsf {T}}_{2j}$ row, $\widehat{\mathsf {T}}_{22} = \widehat{\mathsf {T}}_{23} = 1/2$. Note we use ${\mathscr M}_8$ of Fig. 1 in our analysis.

To illustrate the construction of a triplet transition matrix, we use the simpler ${\mathscr M}_{4} = \left\{ m_{0}, m_{1}, m_{2}, m_{3} \right\}$. Note that the subscripts of $\mathsf {T}$, $i,j = 0,1,2,3$ correspond to subscripts of states $m_i$ in ${\mathscr M}_4$ shown in Fig. 1. An example of the evolution of a network of 5 nodes and the triplet transition matrix is shown in  Fig. 2. Note we will use labelled subgraphs and ${\mathscr M}_8$ for our analysis.

## Evidence for higher order interactions

To investigate the effectiveness of three-node interactions, we start by considering a ‘pairwise model’ whose dynamics is driven only by pair-wise relationships. This will act as a null model when analysing the transition matrix for artificial and real-world networks.

### A pairwise model

  Our ‘pairwise model’ is a stochastic graph model for dynamic networks^50^ in which the evolution of the links is based on comparison of the evolution of the edge between pairs of nodes, so a two-node graphlet model. In the pairwise model, moving from one network, ${\mathscr G}(s)$ to the next network in the sequence, ${\mathscr G}(s+1)$, any pair of nodes with no existing link gains a link with probability *p* otherwise with probability $(1-p)$ the pair of nodes remains unconnected. Similarly, every existing link $e \in ~{\mathscr E}(s)$ is removed with probability *q* otherwise with probability $(1-q)$ the link remains. The number of links is not preserved in this model. This null model is a lower order description of the local interactions than our full analysis in terms of triplets. It is straightforward to write down the form of the transition matrix in our triplet based analysis when assuming this pairwise model gives a precise description, giving us a two-parameter transition matrix denoted as $\mathsf {T}^{({\mathrm {pw}})}(s)$. The detailed calculation of the form is given in Appendix B.

If we assume that graph evolution follows this pairwise mechanism, we can estimate values $\hat{p}(s)$ and $\hat{q}(s)$ for the parameters *p* and *q* respectively by looking at how links changed over one time step, i.e., from the edge set ${{\mathscr E}}(s-1)$ in ${\mathscr {G}}(s-1)$ to edge set ${\mathscr E}(s)$ of ${\mathscr {G}}(s)$. Formally we have that

5 $$\begin{aligned} \hat{q}(s)= & {} 1- \frac{|\mathscr {E}(s-1) \cap {\mathscr E}(s)|}{|{\mathscr E}(s-1)|} \, , \end{aligned}$$

6 $$\begin{aligned} \hat{p}(s)= & {} \frac{\left| {\mathscr E}(s) \setminus ({\mathscr E}(s-1) \cap {\mathscr E}(s) ) \, \right| }{N(N-1)/2 - |{\mathscr E}(s-1)|} \, . \end{aligned}$$

This gives us our pairwise model prediction for the triplet transition matrix $\widehat{\mathsf {T}}^{\mathrm {(pw)}}(s)$, where we substitute $\hat{p}(s)$ from (6) and $\hat{q}(s)$ from (5) for *p* and *q* in $\mathsf {T}^{({\mathrm {pw}})}$ in equation (B1) of appendix B. That is $\widehat{\mathsf {T}}^{\mathrm {(pw)}}(s) = \mathsf {T}^{({\mathrm {pw}})}(\hat{p}(s),\hat{q}(s))$ where

7 $$\begin{aligned} \mathsf {T}^{({\mathrm {pw}})}(p,q) \! = \!\! \begin{pmatrix} (1\!\!-\!p)^3 &{} 3p(1\!\!-\!p)^2 &{}3p^2(1\!\!-\!p) &{} p^3 \\ q(1\!\!-\!p)^2 &{} (1\!\!-\!q)(1\!\!-\!p)^2+2qp(1\!\!-\!p) &{} 2(1\!\!-\!q)p(1\!\!-\!p)+qp^2 &{} (1\!\!-\!q)p^2 \\ q^2(1\!\!-\!p) &{} 2(1\!\!-\!q)q(1\!-\!p)\!+\!q^2p &{} (1\!\!-\!\!q)^2(1\!-\!p)+2qp(1\!-\!q) &{} (1\!-\!q)^2p \\ q^3 &{} 3(1\!-\!q)q^2 &{} 3q(1\!-\!q)^2 &{} (1\!-\!q)^3 \\ \end{pmatrix} \, . \end{aligned}$$

### Quantifying non pairwise interactions

### Real world data sets

The first data set is a Turkish Shareholder network^51^. The nodes are shareholders in Turkish companies and two shareholders are linked if they both hold shares in the same company in one year. The snapshots in the Turkish companies temporalShareholder network, ${\mathscr G}(s)$, are for consecutive years. The second data set is a Wikipedia Mathematicians network. Nodes are pages corresponding to biographies of mathematicians in Wikipedia and two nodes are connected in one snapshot ${\mathscr G}(s)$ if there is a hyperlink (in either direction) between the two pages at the time the data is taken^52^. The third temporal network is constructed from college message data. In this case the nodes are students and an edge in a snapshot indicates that the students exchanged a message within the interval associated with that snapshot^53–55^. We also use a network based on face-to-face contacts at a conference on hypertext. In the network, a node represents a conference visitor, and an edge represents a face-to-face contact that was active for at least 20 s^56,57^. Finally the Email network is derived from the emails at a large institution. Each node corresponds to an email address. An edge in a given snapshot indicates that an email was sent between the nodes in the time interval corresponding to that snapshot^53,58,59^. For temporal networks based on real data, the actual time interval between consecutive graphs in our temporal network, between ${\mathscr G}(s)$ and ${\mathscr G}(s+1)$, is given in real units as $\Delta t$. In some data sets we are able to look at the same data with different real time intervals between data sets. We provide a summary of the graph statistics in Table 1:

**Table 1:** Summary of network statistics.

Dataset	(Abbreviation)	Nodes (*N*)	Edges in static graph (*E*)	Temporal edges (*TE*)
Wikipedia mathematicians	(WikiMath)	6049	15016	36315
Turkish shareholder	(Shareholder)	1804	20981	27218
Hypertext	(Hypertext)	113	2196	20818
College message	(CollegeMsg)	1899	20296	59835
Institution email	(Email)	986	24929	332334

Edges in static graph gives the number of node pairs that have at least one interaction while the Temporal Edges gives the total number of interactions between edge pairs, so some edge pairs have more than one temporal edge.

### Quantifying non pairwise interactions

The pairwise model captures the effects of the interaction of node pairs. We can use this in the form $\widehat{\mathsf {T}}^{\mathrm {(pw)}}(s)$ in (7) as a benchmark to show how higher-order information is captured by our triplet based analysis using $\widehat{\mathsf {T}}(s)$ of (4). One way to study this for any given temporal network is to look at the difference $\Delta \widehat{\mathsf {T}}(s)$ of the transition probabilities between the empirical triplet transition and the assumed pairwise null model:

8 $$\begin{aligned} \Delta \widehat{\mathsf {T}}(s) = \widehat{\mathsf {T}}(s) - \widehat{\mathsf {T}}^{\mathrm {(pw)}}(s), \end{aligned}$$

where each entry in the matrix represents the difference between the estimated triplet transition probability and pairwise transition probability for different states.

To test our approach using our triplet transition matrices, we first look at artificial temporal graphs created from three simple models. The first model, the pairwise model, is simply the same pairwise interaction mechanism used above to define $\widehat{\mathsf {T}}^{\mathrm {(pw)}}(s)$. The second model, the edge swap model, is the configuration model which is also based on pairs of nodes. The third model, the random walk model, uses short random walks in order to find new target nodes when rewiring an edge, and so this involves higher-order interactions. In the last two models, a fraction of edges in ${\mathscr G}(s)$ are rewired to give the next graph ${\mathscr G}(s+1)$ in the temporal network. The results are as expected with matrix $\Delta \widehat{\mathsf {T}}(s)$ close to zero for all entries for the first two models based on node pairs and it is only with the third higher-order model that some $\Delta \widehat{\mathsf {T}}(s)$ entries are found to be large. The networks constructed are undirected. The details of the models and of the results are given in Appendix B.

**Figure 3:**
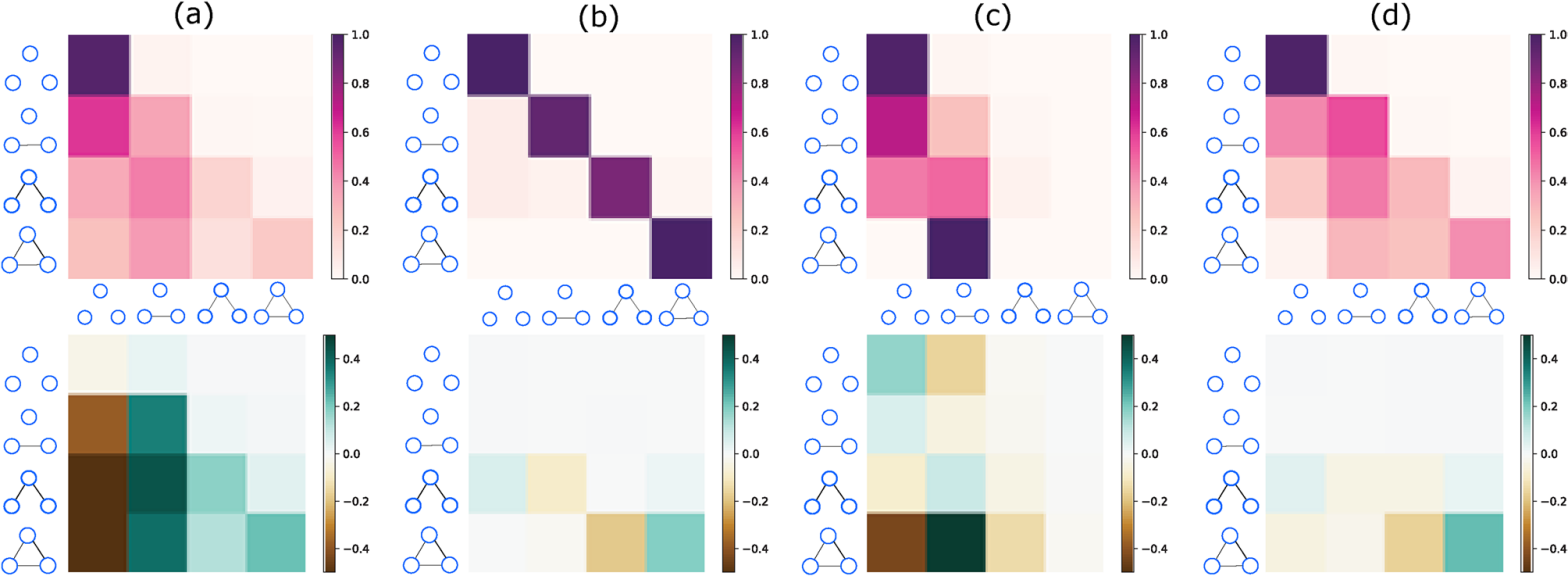
The Triplet transition matrix $\widehat{\mathsf {T}}$ evaluated for real world networks. The average values $\langle \widehat{\mathsf {T}} \rangle$ (4) are shown on the top row while the average of differences from the simple pairwise model, $\langle \Delta \widehat{\mathsf {T}} \rangle$ of (8), are shown on the bottom row. Each of the four-by-four heat map grids is organised in the same way as the $\widehat{\mathsf {T}}$ matrix shown in Fig. 2. That is, the rows indicate the initial triplet state ($m_0$ to $m_3$ from top to bottom as indicated) and the columns indicate the final triplet state ($m_0$ to $m_3$ from left to right as indicated). The values of $\langle \widehat{\mathsf {T}} \rangle$ on the top row run from 0 (white) to 1.0 (dark red). For $\langle \Delta \widehat{\mathsf {T}}(s) \rangle$ on the bottom row, values run from dark brown (-0.5) through white (0.0) to dark blue ($+0.5$). Each column of heat maps is for a different data set; from left to right we have: (a) Turkish Shareholder networks ($\Delta t = 2 {\mathrm yr}$), $p=3.49\times 10^{-2}$, $q=0.638$, (b) Wikipedia Mathematicians networks ($\Delta t = 1 {\mathrm yr}$), $p=5.71\times 10^{-4}$ $p=3.19\times 10^{-4}$, $q=0.068$, (c) College Message networks ($\Delta t = 1 {\mathrm mo}$), $p=0.067$, $q=0.510$, and (d) Email networks ($\Delta t = 1 {\mathrm mo}$), $p=9.43\times 10^{-3}$, $q=0.432$. The actual time difference between snapshots ${\mathscr G}(s)$ and ${\mathscr G}(s+1)$ is $\Delta t$.

We then apply this framework to analyse some real data sets. For clarity we show results for our triplet transition matrix (8) when working with ${\mathscr M}_4$ and these are shown in Fig. 3. Not surprisingly, given these are from real world data sets, these who significant non-zero entries in our measure $\Delta \widehat{\mathsf {T}}(s)$ but also they all look very different from each other, reflecting different mechanisms behind the evolution of different systems.These results for real-world systems have significant non-zero entries in our measure $\Delta \widehat{\mathsf {T}}(s)$. These results for $\Delta \widehat{\mathsf {T}}(s)$ are also very different from each other, reflecting distinct mechanisms behind the evolution of these systems.

When we look for higher order interactions, we find clear differences between the triplet transition matrix $\widehat{\mathsf {T}}$ and the simple pairwise reference model of $\widehat{\mathsf {T}}^{\mathrm {(pw)}}$, especially in the Turkish Shareholder network Fig. 3a. For example, compared to the corresponding probability in the pairwise model, the real probability of any triplet state becoming disconnected in the next snapshot (i.e., moving from $m_i \rightarrow m_0$, $i \in \{0,1,2,3\}$, the first column of the transition matrix) is much less, showing that this subgraph is very stable compared with the pairwise case in the Turkish Shareholder network. Additionally, the state is much more likely to evolve to $m_1$ at $s+1$ (the second column of the transition matrix), which demonstrates that in many real networks, the interactions are beyond the pairwise interaction. The significance test using Z-score can be found in Appendix D.

In our analysis of real data, we use $\Delta t$ to denote the physical time difference between snapshots ${\mathscr G}(s)$ and ${\mathscr G}(s+1)$. This time interval can be varied when working with the College message data of Fig. 3c and the Email networks of Fig. 3d. In these cases we try several values of $\Delta t$ before choosing an appropriate value the the illustrations in Fig. 3. We are looking for a value that is not too short (nothing much happens) and not too long (averaging over uncorrelated changes).

Unsurprisingly, all four real world networks show considerable higher-order effects but there are interesting differences that reveal the processes behind the evolution are likely to have some significant differences. The $\widehat{\mathsf {T}}$ results are shown in the top row of Fig. 3. The result for the Turkish Shareholder networks in Fig. 3a and the Email networks in  Fig. 3d look very similar. However, when we compare them to our reference model, the pairwise model, we see large differences, showing that these are very different types of temporal network and using raw values of $\widehat{\mathsf {T}}$ can be misleading.

In fact, the networks which are most similar, once simple pairwise processes are discounted, are the Wikipedia Mathematicians networks Fig. 3b and the Email networks  Fig. 3d. In both cases, processes where an edge is added (upper triangle in the heat map) there is little difference from the pairwise model. This might be expected for the cases where there is at most one edge in the triplet. Only the $\Delta \widehat{\mathsf {T}}_{23}$ entry for the $m_2 \rightarrow m_3$ transition shows a slight increase over what would be predicted based on the rate of edge addition in these models (the *p* parameter of the pairwise model) which suggests triadic closure^7,8,47,48^ does play a role here but it is very slight by these measures. On the other hand, there is a strong sign of “triadic stability”, that is the complete graphlet $m_3$ is much more stable than we would expect given the rate of edge loss in the pairwise model (the *q* parameter). So it seems the social processes behind the ideas of triadic closure are possibly more important for the stability of triangles in the contexts of our Wikipedia Mathematician and Email network.It is natural to think that processes responsible for triadic closure ($m_2 \rightarrow m_3$) would also slow the rate at which such triangles break up ($m_3 \rightarrow m_2$) but we do not see this in our result. One interpretation of our results is that the social processes normally invoked for triadic closure^7,8,47,48^ can, in some cases, be more important in preventing the breakup of triangles than in the creation of triangles.

In some ways the similarity between the Wikipedia Mathematicians networks Fig. 3b and the Email networks Fig. 3d is surprising as we might have expected the greatest similarity between the two communication networks, the College Message networks Fig. 3c and the Email networks Fig. 3d but that is not what we see in our measures. In particular, the stability of the complete triangle in the college message is exactly as we would expect based on pairwise measures suggesting different properties in these two communication networks, e.g.i.e. that many of the college messages are between actors who do not have strong ties.

However, the main message in Fig. 3 is that in almost any data, we find the evolution has clear signals of higher-order interactions playing an important role.

## Link prediction

The analysis of our transition matrix of triplets reveals that non-pairwise interaction exists in network evolution. We now ask if these higher-order interaction patterns are essential for network evolution. We investigate this question by performing link predictions for dynamic networks based on the higher-order information stored in our triplet transition matrices.

### Triplet transition score

The idea behind our algorithm is that the formation or removal of links between a node pair is encoded in our triplet interactions. The likelihood of a link appearing (or disappearing) between a node pair in a snapshot can be obtained by looking at the triplets containing this node pair and using the triplet transition matrix to see what that suggests about the evolution of any edge between our chosen node pair.

To keep our analysis simple we will assume that the interval between snapshots $\Delta t$ is about the same size as the appropriate time scale for changes in the network. If we look at snapshots covering a short period of time, nothing much happens and the transition matrix has too little information in it. One could then look at larger times scales by using $\widehat{\mathsf {T}}(s)$, $\widehat{\mathsf {T}}(s-1)$, $\widehat{\mathsf {T}}(s-2)$ and so forth to predict links in the next network snapshot ${\mathscr G}(s+1)$. WeIf we look at snapshots covering an extremely short period of time, say one that covers the typical difference in time between events, then the transition matrix contains too little information. In such a case one could then look at larger times scales by using $\widehat{\mathsf {T}}(s), \widehat{\mathsf {T}}(s-1), \cdots , \widehat{\mathsf {T}}(s-n)$ (for some *n*) to predict links in the next network snapshot ${\mathscr G}(s+1)$. However, we will use the simpler assumption that working with one snapshot by choosing $\Delta t$ appropriately (where we have that choice) captures the essential information. This implies we have avoided the opposite problem, where $\Delta t$ is too large so the information in the transition matrix is averaging over mostly uncorrelated and unrelated events which means we have very little signal encoded in our transition matrices.

Our second assumption is that the transition depends only on the state of the system at the last time step, which means we assume the process is Markovian. In some ways this need not be completely true. Provided the snapshots ${\mathscr G}(s-1)$ and ${\mathscr G}(s)$ are in a similar state to the one we are trying to predict, ${\mathscr G}(s+1)$, then because we are constructing our transition matrices based on the similar states, the history of the evolution may well be encoded in this. For instance, patterns of activity in an email network of a large institution could well change if there is a major reorganisation with many people changing roles or locations in which case the history of the system has an impact on the evolution. Put another way, we assume that any non-Markovian behaviour is happening on much larger time scale than we are studying and so we can use a Markovian approximation.

Our link prediction algorithm assigns a score to each node pair. By looking at the distribution of these scores we can separate them into node pairs with low scores, predicting no link in the next snapshot, or a high score meaning this node pair will be connected. For a given snapshot ${\mathscr G}(s)$, for each node pair, say (*u*, *v*), we look at all $(N-2)$ triplets containing that node pair count the number of each triplet falling in each state $m \in {\mathscr M}$. Normalising this gives us our node-pair state vector $\psi (u,v;s)$ as follows

9 $$\begin{aligned} \psi _i(u,v;s) = \frac{1}{N-2} \sum _{w \in {\mathscr V}\setminus \{ u,v\}} \delta (\mathsf {M}_s(u,v,w),m_i) \, , m_i \in {\mathscr M}\, . \end{aligned}$$

Our estimate for the transition matrix $\widehat{\mathsf {T}}(s)$, based on ${\mathscr G}(s-1)$ to ${\mathscr G}(s)$ evolution, is to be used to tell us about the evolution of this triplet state distribution $\psi (s)$ in (9). For instance we estimate that the probability $L_\beta$ that a pair of nodes *u*, *v* has an link, $\beta =1$, or no link, $\beta =0$, in the graph ${\mathscr G}(s+1)$ is given by the projection from predicted $\psi (s)$:

10 $$\begin{aligned} L_\beta (u,v;s+1) = \sum _{i,j} \psi _i(u,v;s) \, \widehat{{T}}_{ij}(s) \, P^{\mathrm (out)}_{j\beta }, \end{aligned}$$

where $P^{\mathrm (out)}_{j\beta }$ is a projection vector for the triplet evolution and $\sum _{\beta =0}^{1} P^{\mathrm (out)}_{j\beta }=1$. With the other definition $\sum _{j} \widehat{\mathsf {T}}_{ij} =1$, and $\sum _{i} \psi _i(u,v;s)=1$, then we have that $L_\beta$ is properly normalised $\sum _{\beta =0}^{1} L_\beta (u,v) =1$. It is this $L_\beta$ in (10) that we use as a score for link prediction.

Take the case of ${\mathscr M}_4$ as an example, the projection from triplet distribution onto links uses

11 $$\begin{aligned} {{\mathbf {\mathsf{{P}}}}}^{\mathrm (out)}= \begin{pmatrix} 1 &{} 0 \\ 2/3 &{} 1/3 \\ 1/3 &{} 2/3 \\ 0 &{} 1 \\ \end{pmatrix}. \end{aligned}$$

The factors here arise for the state set ${\mathscr M}_4$ since the unlabelled graphlets do not distinguish which links are occupied in the one-link triplet $m_1$ or two-link triplet $m_2$. For the state set ${\mathscr M}_8$ which we use in most of our work, this projection matrix is much simpler with entries either 0 or 1. If we choose (*u*, *v*) to be the first link, so it is the only link in the triplet we call $n_1$ in Fig. 1, then we can use bitwise logical operators to represent ${{\mathbf {\mathsf{{P}}}}}^{\mathrm (out)}_{j\beta }$ as $(j \; {\mathrm AND} \; 1)\; {\mathrm XOR} \; \beta$.

### Node similarity

We are using link prediction as a way to test that our triplet transition matrices $\widehat{\mathsf {T}}$ capture important higher-order interactions in the evolution of temporal networks. In order to see how effective our approach is, we need to compare against other methods of link prediction. All the methods we use are listed in Table 2. All these methods can be discussed in terms of a node similarity score and in this section we will start our examination of these methods by considering the node similarity scores used in each method. This will allow us to examine the relationships between these various methods and ask if they capture higher-order interactions to any extent. The next stage is to turn these similarity scores into a link prediction and we will look at this step in the next section.

**Table 2:** Table of the link prediction methods used and their abbreviations.

Abbreviation	*Method*${}^{\text {Reference}}$	Length scale	Code
AAI	*Adar-Academic Index*^60^	2	nx
CN	*Common neighbour*^60^	2	nx
EE	*Edge existence*	1	-
JC	*Jaccard Coefficient*^60^	2	nx
Katz	*Katz*^61^	$\infty$	own
LLHN	*Local Leicht–Holme–Newman*^62^	2	own
LPI	*Local path Index*^63^	3	own
MFI	*Matrix Forest Index*^64^	$\infty$	own
PA	*Preferential attachment*^60^	2	nx
RAI	*Resource allocating Index*^63^	2	nx
TT	*Triplet transition*	$\infty$	own

The length scale given indicates the longest path length involved in the method or equivalently the largest power of the adjacent matrix involved in the method. Under code, nx indicates that a NetworkX^65^ library routine was used, while own indicates the authors’ own code was used. The Edge Existence (EE) approach was not investigated numerically but was included for the sake of comparison. All these methods, except for the Triplet Transition method, have a temporal path length of 0. That is they are derived solely from the current snapshot, ${\mathscr G}(s)$, when making a prediction for links in the next snapshot ${\mathscr G}(s+1)$. The Triplet Transition method has a temporal path length of 1 as it uses ${\mathscr G}(s)$ and ${\mathscr G}(s-1)$ to predict ${\mathscr G}(s+1)$.

The various link prediction methods can be categorised based on the type of information used to make a prediction about each node pair: those which use only local interactions probing a fixed distance from the node pair, and those global methods which use nodes arbitrarily far from the node pair of interest. This is indicated by the length scale of methods indicated in Table 2 and will be clear from the power of the adjacency matrix ${{\mathbf {\mathsf{{A}}}}}$ appearing in the similarity scores defined in this section.

**Figure 4:**
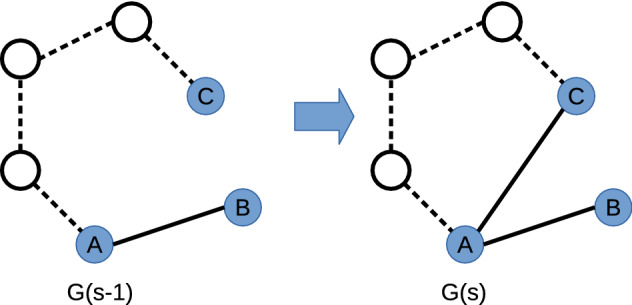
An illustration of how information on long range paths between nodes plays a role in our Triplet Transition method. This shows the evolution of one $m_1$ triplet in snapshot $G(s-1)$ to become an $m_2$ triplet in the next snapshot. The triplet nodes are shown as solid blue circles connected by solid black edges. The network outside the triplet is shown with nodes as open circles connected by edges shown as dashed lines. It is the network outside the triplet which provides a long distance connection between triplet nodes *A* and *C*. Within the original *ABC* triplet in snapshot $G(s-1)$, *C* appears disconnected. By using two snapshots, our transition matrix includes the effects of such long-range links and so this approach can explain why the edge (*A*, *C*) might be appear more often than otherwise expected from the triplet subgraph alone.

One other point can be made. In terms of the temporal network, none of these existing similarity scores, none of the these link prediction methods from the literature, use more than one temporal snapshot ${\mathscr G}(s)$. So one standout feature of our method is the use of two temporal snapshots, ${\mathscr G}(s)$ and ${\mathscr G}(s-1)$. The evolution of a triplet from one snap shot to another records, in an indirect way, the effects of other nodes beyond the triplet of interest as illustrated in Fig. 4. This is why our method is listed as probing long length scales in Table 2. So one standout feature of our method is the use of two temporal snapshots, ${\mathscr G}(s)$ and ${\mathscr G}(s-1)$. The evolution of a triplet from one snap shot to another records, in an indirect way, the effects of other nodes beyond the triplet of interest. This is why our method is listed as probing long length scales in Table 2.

In the following *s*(*u*, *v*) is a (similarity) score assigned to a pair of vertices $u,v \in {\mathscr V}$ where ${\mathscr N}(u)$ is the set of neighbours of vertex *u*, i.e. ${\mathscr N}(u) = \{ w | (u,w) \in {\mathscr E}\}$. The similarity scores are then used to decide if an edge should exist between *u* and *v* and that will be used in turn to make the link prediction for the next snapshot in time. We will assume a simple graph in these discussions.

The simplest node similarity measure is the *Edge Existence* (EE) index $s_{\mathrm EE} (u,v)$. After all, if there is already an edge between two vertices *u* and *v* this is a good indication of a close relationship between these nodes, and vice versa. We define Edge Existence index to be

12 $$\begin{aligned} s_{\mathrm EE} (u,v) = A_{uv} = \sum _{e \in {\mathscr E}} \delta _{e,(u,v)}. \end{aligned}$$

That is Every edge between *u* and *v* adds one to this index so, for a simple graph, this is the corresponding entry of the adjacency matrix, $A_{uv}$. This is the simplest edge prediction method in that it predicts no changes at all so it is not very useful and we do not use it in our work. However, we will see this score contributes to some of the other, more sophisticated methods, so it is useful to define this score. The Edge Existence score records no higher-order effects, there is a path of length at most one between the nodes.

While not very useful, this *Edge Existence* (EE) index could produce some very good statistics. For instance, if very few edges are changing in each time interval, then this method would predict the behaviour of the vast majority of edges as most remain unchanged. It would only do badly if we used statistics that specifically measured the success of node pairs changing their connectedness from one time step to the next. A good example ofthis when the Edge Existence method would appear to be successful is when we break up the data into small steps in time where few edges change. However we would learn little of interest in this case. would be when we are able to break the data up into arbitrarily sized steps in time, and if we choose steps which are too small, we will get little change step because of this discretisation choice.

The *Common Neighbours* (CN) method^60,61,66^ simply scores the relationship between two nodes based on the the number of neighbours they have in common

13 $$\begin{aligned} s_{\mathrm CN}(u,v) = \sum _{w\in {\mathscr V}} A_{uw} A_{wv} = | {\mathscr N}(u) \cap {\mathscr N}(v) | \, . \end{aligned}$$

This will tend to give large scores if *u* and/or *v* have high degrees and this is illustrated in more detail in Appendix E.

One way to compensate for the expected dependence of $s_{\mathrm CN}$ on the degree of the nodes is to normalise by the total number of unique neighbours. This gives us the *Jaccard Coefficient*^60,66^ (JC) method based on the well known similarity measure^68^ in which the likelihood that two nodes are linked is equal to the number of neighbours they have in common relative to the total number of unique neighbours.

14 $$ s_{{{\text{JC}}}} (u,v) = \frac{{|{\mathscr{N}}(u) \cap {\mathscr{N}}(v)|}}{{|{\mathscr{N}}(u) \cup {\mathscr{N}}(v)|}} = \frac{{s_{{{\text{CN}}}} (u,v)}}{{k_{u}  + k_{v}  - 2s_{{{\text{EE}}}} (u,v) - s_{{{\text{CN}}}} (u,v)}}. $$

The *Preferential Attachment* (PA) method for link prediction^60,66^ is based on the idea that the probability of a link between two vertices is related to the product of their degrees of the two vertices

15 $$ s_{{{\text{PA}}}} (u,v) = |{\mathscr{N}}(u)|\cdot  |{\mathscr{N}}(v)|. $$

This is proportional to the number of common neighbours expected in the Configuration model^69^ as has been used in the context of collaboration (bipartite) collaboration graphs^60,70,71^.

The *Resource Allocating Index*^63^ (RAI) method and the *Adamic-Adar Index*^60,66^ (AAI) method are both based on the idea that if two vertices *u* and *v* share some ‘features’ *f* that is very common in the whole network then that common feature is *not* a strong indicator that the two vertices should be linked. The converse is true if the common feature is rare, that is a good indicator that the vertices *u* and *v* should be linked. So in general if *W*(*x*) is a monotonically decreasing function of *x* we can use this on the frequency *n*(*f*) of the occurrence of feature *f* to give a generic similarity function of the form $s_{\mathrm W}(u,v) =\sum _{f \in u, v} W(n(f))$. In our case, we will not assume any meta-data exists, but we will look for methods that use features which are based purely upon the topology of the network.

In the Resource Allocating Index^63^ the inverse of the degree of the neighbour is used as the weighting function, so $W(n(f)) \equiv 1/|{\mathscr N}(w)|$ giving

16 $$\begin{aligned} s_{\mathrm RAI}(u,v) =\sum _{w \in {{\mathscr N}(u) \cap {\mathscr N}(v)} } \frac{1}{|{\mathscr N}(w)|} \, . \end{aligned}$$

Note that this means that the contribution from any one node *w* to the total of all scores $S_{\mathrm RAI}(u,v) = \sum _{u,v} s_{\mathrm RAI}(u,v)$ is half of the degree of *w* minus one, $({\mathscr N}(w)-1)/2$. Thus the Resource Allocating Index still gives high degree nodes more weight.

On the other hand, the Adamic-Adar Index^60,66^ uses the inverse logarithm of the degree to weight the contribution of each common neighbour to the score, $W(n(f)) \equiv 1/\ln (|{\mathscr N}(w)|)$. That is

17 $$\begin{aligned} s_{\mathrm AAI}(u,v) = \sum _{w \in {{\mathscr N}(u) \cap {\mathscr N}(v)} } \frac{1}{\ln ( |{\mathscr N}(w)| ) } \, . \end{aligned}$$

All the indices mentioned above have been based either on the degree of the two nodes of interest $s_{EE}$ or on the properties of *u*, *v* and their common nearest neighbours $w \in {\mathscr N}(u) \cap {\mathscr N}(v)$. This involves paths between the two nodes *u* and *v* of length two or less. The next logical step is to include paths of length three and the *Local Path Index* (LPI) $s_{\mathrm LPI}$^61,63^ is an example of this where

18 $$\begin{aligned} s_{\mathrm LPI}(u,v) = [{{\mathbf {\mathsf{{A}}}}}^2 + \beta {{\mathbf {\mathsf{{A}}}}}^3]_{uv}= s_{\mathrm CN}(u,v) + \beta [{{\mathbf {\mathsf{{A}}}}}^3]_{uv} \, . \end{aligned}$$

Here $\beta$ is a real parameter where $\beta =0$ reproduces the Common Neighbours score of (13). The $\beta {{\mathbf {\mathsf{{A}}}}}^3$ term is counting the number of walks of length three that start at *u* and end at *v*. If there is already an edge between *u* and *v* then $[{{\mathbf {\mathsf{{A}}}}}^3]_{uv}$ includes backtracking paths such as the sequence *u*, *w*, *u*, *v*. This means the second term also includes a term equal to $A_{uv}(|{\mathscr N}(u)|+|{\mathscr N}(v)|)$, i.e. there is a contribution from the Edge Existence similarity $s_{\mathrm EE} (u,v)$ (12) in this method.

The *Katz Index*^60,61,66^ (Katz) counts the number of paths between each pair of vertices, where each path of length $\ell$ contributes a factor of $\beta ^\ell$ to the score. The score is simply the appropriate entry of the matrix $[{{\mathbf {\mathsf{{I}}}}}-\beta {{\mathbf {\mathsf{{A}}}}}]^{-1}$,

19 $$\begin{aligned} s_\text {Katz}(u,v) = ([{{\mathbf {\mathsf{{I}}}}}-\beta {{\mathbf {\mathsf{{A}}}}}]^{-1})_{uv} \, , \end{aligned}$$

where $\beta$ is positive but must be less than the largest eigenvalue of the adjacency ${{\mathbf {\mathsf{{A}}}}}$. Note for low $\beta$ and for a simple graph we have that

20 $$\begin{aligned} s_{\mathrm Katz}(u,v) = \beta A_{uv} + \beta ^2 \sum _w A_{uw}A_{wv} + \beta ^3 \sum _{w,x} A_{uw}A_{wx}A_{xv} + O(\beta ^4) = \beta s_{\mathrm EE}(u,v) + \beta ^2 s_{\mathrm LPI}(u,v) + O(\beta ^4) \quad \text{ for } u \ne v \end{aligned}.$$

The *Local Leicht-Holme-Newman Index*^72^ (LLHN) is based on the vertex similarity index of^62^, but while this gives a specific motivation for the form, it is in the end just a specific rescaling of the Katz index (19), namely

21 $$\begin{aligned} s_{\mathrm LLHN}(u,v) = \frac{s_{\mathrm Katz}(u,v)}{s_{\mathrm PA}(u,v)} = \frac{s_{\mathrm Katz}(u,v)}{|{\mathscr N}(u)| \, |{\mathscr N}(v)|} = {{\mathbf {\mathsf{{D}}}}}^{-1} \left( {{\mathbf {\mathsf{{I}}}}}- \beta {{\mathbf {\mathsf{{A}}}}}\right) ^{-1} {{\mathbf {\mathsf{{D}}}}}^{-1} \end{aligned}$$

where ${{\mathbf {\mathsf{{D}}}}}$ is a diagonal matrix whose entries are equal to the degrees of the nodes, $D_{uv} = \delta _{u,v} |{\mathscr N}(u)|$. The motivation for using this normalisation is that $|{\mathscr N}(u)| \cdot |{\mathscr N}(v)|$ is proportional to the number of neighbours expected in the configuration model, as shown in Appendix E. So the Local Leicht-Holme-Newman Index is the Katz score relative to the Katz score expected for the same pair of nodes in the configuration model.

The *Matrix Forest Index*^64^ (MFI) is defined as:

22 $$\begin{aligned} s_{\mathrm MFI}(u,v) = [( {{\mathbf {\mathsf{{I}}}}}+ {{\mathbf {\mathsf{{L}}}}})^{-1}]_{uv} \, , \end{aligned}$$

where ${{\mathbf {\mathsf{{L}}}}}= {{\mathbf {\mathsf{{D}}}}}- {{\mathbf {\mathsf{{A}}}}}$ is the Laplacian. One way to understand the Matrix Forest Index is to consider the diffusion process described by a Laplacian. If we were to demand that at time $t+1$ we had only had particles at one site *u*, then $s_{\mathrm MFI}(u,v)$ tells us how many of those particles were at vertex *v* at the previous time step.

### From node similarity to link prediction

All the link prediction methods used in this paper, see Table 2, assign a similarity score between pairs of nodes. To turn this into a link prediction, the basic conjecture is that the higher the similarity between a pair of nodes, the more likely we are to find a link between these two nodes.

All methods, therefore, require a precise method to turn the scores into predictions, essentially to define what is meant by a ‘high’ or a ‘low’ score by introducing a *classification threshold*. Often this is done very simply by ranking the scores and using a fixed number of the most highly ranked node pairs to predict a link. This method is typically used only for *link addition* in which one is only trying to predict when an unlinked node pair gains a link, that is the $A_{uv}(s)=0$ to $A_{uv}(s+1)=1$ process.

We are interested in the most general predictions, looking at all four possible changes for node pairs from one snapshot in time to the next, that is all the four possible $A_{uv}(s)=0,1$ to $A_{uv}(s+1)=0,1$ processes. This is *link evolution* rather than link addition. In order to make these more general predictions for any method, we use *k*-means clustering methods to separate the prediction scores produced by each method into two classes: a high score group and a low score group of node pairs. Any node pair with a score in the high scoring group will be predicted to have a link in the next snapshot; node pairs in the low scoring group will be predicted to have no link.

**Figure 5:**
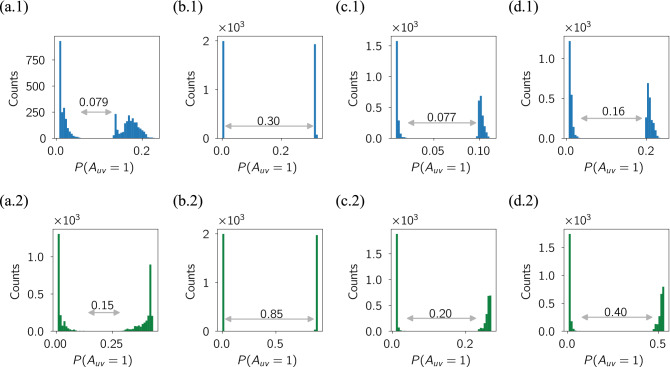
The histogram of node similarity scores in our triplet transition (TT) method for (**a**) Turkish Shareholder networks, (**b**) Wikipedia Mathematician networks, (**c**) College Message networks, and (**d**) Email networks. The precise values are not important as the important feature is the clear separation of the node similarity scores into two groups. One cluster $c_0$ appears to have a ‘low’ probability that a link will exist in the next snapshot and the other cluster has ‘high’ probability. We therefore predict that if the node pair has a score in the lower score cluster $c_0$, then a link will not exist in the next snapshot. Conversely, if a node pair exists in the higher score cluster $c_1$, then we predict a link will exist between this node pair in the next snapshot. The first row, where the histogram is plotted in blue is the clustering results for the ${\mathscr M}_4$ while the second row, where the histogram is plotted in green is the clustering results for the ${\mathscr M}_8$. It shows that the ${\mathscr M}_8$ has higher separation between two clusters than ${\mathscr M}_4$. The results for each network are based on samples which contain at least 2000 node-pairs with an initial link and at least 2000 more node pairs which started without a link.

To show how this works, we give some examples of how the score from our triplet transition method produces a natural split into low and high scores which is easily discovered by an automated clustering method such as *k*-means, as seen in the first-row of Fig. 5. In our final results below for our link prediction algorithm, we use ${\mathscr M}_8$ and the clear separation of node similarity scores into a low and high group is shown in the second row of Fig. 5. For comparison we also show the results for our method using the less sensitive ${\mathscr M}_4$. In practice, both seem to separate low and high scoring node pairs well but we get a slightly clearer separation in some cases when using ${\mathscr M}_8$.

**Figure 6:**
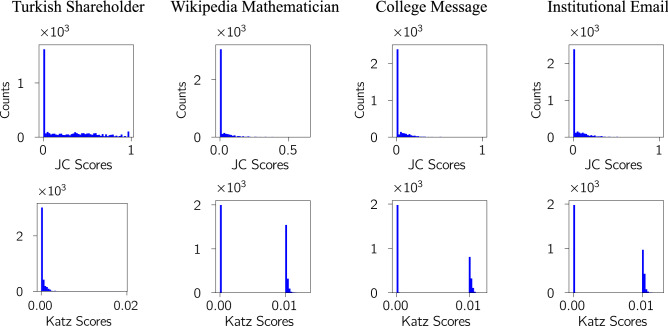
The histogram of node similarity scores using Jaccard Coefficient (JC) & Katz methods on four real data sets. Each column is for one data set which are, from left to right: Turkish Shareholder networks, WikiWikipedia Mathematician networks, College Messages network, and Email networks. The first row is the similarity score used in Jaccard Coefficient method and the second is for the Katz method. The precise values are not important here as the key feature is the success or failure to identify two clear groups of low- and high-scoring node pairs. We under-sampled 1000 linked node pairs and 1000 non-connected node pairs^67^. Only the Katz method (bottom row) shows the necessary clustering of scores needed for link prediction. Similar plots for all the link prediction methods used in this paper are shown in Figure E11 of the Appendix.

We can look at how the other nine link prediction methods perform in terms of identifying two clear groups of low- and high- scoring node pairs. What is surprising is that most of the algorithms fail to do this well, or some cases at all, in most of the data sets as shown in Section E of the Appendix. An example of a poor separation, the Jaccard Coefficient method, is shown in Fig. 6. The one exception is the Katz method, also shown in Fig. 6, which works well in three of the four data sets. Given this is one of the methods probing large distances in the network, this would seem to support the idea that higher-order interactions are important in link prediction.

Since our triplet transition method splits the node similarity scores so well, any unsupervised clustering method should be able to split the node pairs into a low scoring cluster and a high scoring cluster. As the problem is in one-dimension a version of k-means clustering is sufficient and this will always assign our node pairs to either a low or to a high score even if a method does not have a clear threshold visually. The objective function of our clustering is *J* where

23 $$\begin{aligned} J (s_{\mathrm th}) = \sum _{(u,v)} \Big [ \theta \big (s_{\mathrm th} - s(u,v) \big ) \cdot \big |s(u,v) -\mu _- \big | \, + \, \theta \big (s(u,v)- s_{\mathrm th} \big ) \cdot \big |s(u,v) -\mu _+ \big | \, \Big ] \, , \end{aligned}$$

where $\theta$ is the Heaviside step function. The sum over (*u*, *v*) is over all distinct nodes pairs (so $(u,v) \in {\mathscr V}^2 | u \ne v$). The $\mu _+$ ($\mu _-$) is the average similarity score of node pairs in the high score (low score) cluster. The problem is reduced to finding the threshold value $s_{\mathrm th}$ that minimises *J*.

### Evaluation metrics

To evaluate the performance of our Triplet Transition and the other link prediction methods, we use a variety of standard metrics.The simplest metric we use is to simply look at the fraction of edges that are removedThe simplest metric we use is the fraction of edges that are removed from snapshot *s* to the next snapshot $s+1$. We denote this by $f_{\mathscr E}(s)$,

24 $$\begin{aligned} f_{\mathscr E}(s) = 1 - \frac{|{\mathscr E}(s+1) \cap {\mathscr E}(s)|}{|{\mathscr E}(s)|} \, , \end{aligned}$$

where ${\mathscr E}(s)$ is the link set of the network in snapshot *s*.

We also use more traditional metrics such as precision and area under the curve for which we need a binary classification. Thinking of snapshot ${\mathscr G}(s+1)$ as the current state while we are trying to predict the state in the next snapshot, we consider four transitions $(A_{uv}(s) \rightarrow A_{uv}(s+1) )$ of node pairs: $0 \rightarrow 0$, $0 \rightarrow 1$, $1 \rightarrow 0$, and $1 \rightarrow 1$. We map these states onto a binary set of states, namely $A_{uv}(s+1)$, so what we call a positive result (negative result) is where a link is present (is not present) in snapshot $(s+1)$ regardless of the state that pair started in. We then consider whether the prediction for the state of the node pair in snapshot $s+1$ was true or false. So a false positive is where we predict no link for a node pair when that pair did end up with a link, while a true negative is where we correctly predict a pair of nodes will not be connected in the next snapshot. The table of predicted classes and actual classes is shown in Table 3.

**Table 3:** The Confusion matrix for the prediction of edges between nodes *u* and *v* in the next snapshot, $(s+1)$.

		Actual classes	
		$$A_{uv}(s+1) = 1$$	$$A_{uv}(s+1) = 0 $$
Predicted classes	$A_{uv}(s+1) = 1$	True positive ( $01+, 11+$)	False positive ( $01-, 11-$)
	$$A_{uv}(s+1) = 0 $$	False negative ( $$00-, 10-$$)	True negative ( $$00+,10+$$)

The adjacency matrix at snapshot *s* in the temporal network is ${{\mathbf {\mathsf{{A}}}}}(s)$. Each of the four outcomes comes from two situations as this confusion matrix does not consider the state of the edge in snapshot ${\mathscr G}(s)$. The two states are shown for each of outcome in brackets with the notation that $\alpha \beta \pm$ shows the value of $A_{uv}(s)$ as $\alpha$, the predicted $A_{uv}(s+1)$ as $\beta$, and a $+$ symbol (a − symbol) indicates that the prediction made was correct (incorrect).

With this traditional binary classification, we can then evaluate the performance of our Triplet Transition method using standard metrics (see Appendix F for more details)—area under curve and precision. To express these, we define $N_{\alpha \beta \pm }$ to be the number of node pairs satisfying the following criteria. The node pair starts in snapshot ${\mathscr G}(s)$ in state $\alpha$ equal to 1 if the node pair is connected by an edge, 0 otherwise. This edge pair is then in state $\beta$ in snapshot ${\mathscr G}(s+1)$ with $\beta$ is 1 if the node pair is connected by an edge in snapshot ${\mathscr G}(s+1)$, and is 0 otherwise. Finally the sign indicates if the prediction made for that node pair in ${\mathscr G}(s+1)$ was correct (true, $+$) or incorrect (false, −).

We can evaluate the methods independent of the classification threshold chosen through k-means by using the *area under the curve* (AUC) where the curve is the receiver operating characteristic curve. For any binary classifier of the results of an algorithm, such as defined here in Table 3, the curve plotted is the fraction of positive results (TPR, true positive rate) which are correct against the fraction of negative results which are incorrect (FPR, false positive rate) as the threshold $s_{\mathrm th}$ is varied:

25 $$\begin{aligned} {\mathsf TPR} = \frac{N_{11+}+N_{01+}}{N_{11+}+N_{01+}+N_{00-}+N_{10-}} \, , \quad {\mathsf FPR} = \frac{N_{01-}+N_{11-}}{N_{01-}+N_{11-}+N_{00+}+N_{10+}} \, . \end{aligned}$$

Once the threshold $s_{\mathrm th}$ has been fixed, in our case using k-means (23), the *precision* score $S_{\mathrm {prec}}$ is defined as the number of times that we predict a link exists between node pairs in the later snapshot correctly (a true positive, $N_{11+}+N_{01+}$) divided by the number of times we predict a link, correctly (true positive) or incorrectly (false positive, $N_{11-}+N_{01-}$):

26 $$\begin{aligned} S_{\mathrm {prec}}= & {} \frac{ N_{11+}+ N_{01+} }{ N_{11+}+ N_{01+} + N_{11-}+ N_{01-} } \, . \end{aligned}$$

A high precision score means we can trust that links predicted by the algorithm will exist.

We can set a baseline value for precision using a simple model which predicts a link in snapshot ${\mathscr G}(s+1)$ exists for any given node pair with a probability given by the fraction of node pairs which have an link in snapshot ${\mathscr G}(s)$, that is the density $\rho (s)$. In this case, the precision (26) is simply equal to the density in snapshot ${\mathscr G}(s)$, $S_{\mathrm {prec,base}} =\rho (s)$, see Appendix F.

A very low precision score for baseline method indicates that the links existing between node pairs are not random. Some of the local measurement algorithms give lower scores than the baseline, suggesting that those types of local information are less likely to drive the evolution of the networks.

### Edge sampling

We use the whole network to evaluate the performance, which is different from the sampling method used in Fig. 5 to take account of the full nature of the data. In the following analysis, we predict the node pairs for all the possible node pairs of a whole network. We expect predictions can capture evolving characteristics of networks and the Mathematician networks change tiny fractions which are not sufficient enough to evaluate the predictions. Therefore, we replace the Mathematician networks with Hypertext networks (see Appendix C for more details of this data set) in the following prediction analysis. Time data is sampled over different time intervals $\Delta t$. For Turkish Shareholder networks, the smallest time separation is 2 years, and we consider four or more years too long for the evolution; for the Hypertext data, which records the short communications, we choose $\Delta t$ as 40 and 60 minutes; for the College Message and Email networks, we consider that 8 hour to 1 month communication frequency is reasonable.

## Results

**Figure 7:**
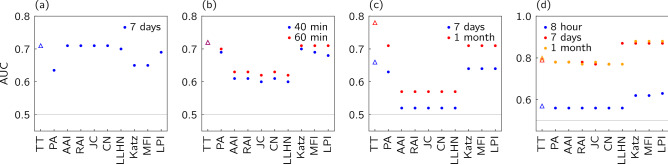
The area under the curve (AUC) results for four temporal networks constructed from real data sets: (**a**) Turkish Shareholder networks, (**b**) Hypertext networks, (**c**) College Message networks, (**d**) Email networks. The results compare ten different methods of Table 2 including our TT algorithm denoted by the ‘triangle’ symbol. For networks (**b**–**d**), we show the results for different time scales (windows). See Table 2 for abbreviations used in indicate link prediction methods.

Figure 7 shows a comparison of AUC for the ten algorithms when we apply them to node pairs sampled uniformly at random from all possible node pairs. The Triplet Transition (TT) approach defined here is the left-most triangular point in each figure. In the Hypertext networks (b) and the College Message networks (c), the Triplet Transition method has the highest AUC though the PA, Katz, MFI and LPI algorithms perform almost as well (see Table 2 for abbreviations). For the Turkish Shareholder network (a) the AUC of our Triplet Transition method is again the highest though with a similar AUC value for various algorithms. Note that PA, Katz, and MFI are now weaker. Only for the Email network (d) is our Triplet Transition outperformed, in this case by the Katz, MFI and LPI algorithms.

**Table 4:** Summary of AUC-ROC performance, $\Delta t$ is taken by selecting the highest AUC-ROC.

Type	Algorithm	Shareholder		Hypertext		CollegeMsg		Email		Avg.
	(Time scale)	(2 years)		(1 h)		(1 mon)		(1 mon)		Rank
	$[\overline{f_\mathscr {E}(s)}]$	[0.63]		[0.52]		[0.93]		[0.50]		
Global	*Triplet Transition*	$\mathbf {0.71}$	1	$\mathbf {0.72\,(7)}$	1	$\mathbf {0.77\,(6)}$	1	$0.80\,(2)$	4	1.8
	*Katz* ($\beta =0.01$)	0.65	8	$\mathbf {0.71\,(10)}$	1	$0.69\,(8)$	2	$\mathbf {0.88\,(2)}$	1	3.0
	*Matrix Forest Index*	0.65	8	$\mathbf {0.71\,(9)}$	1	$0.69\,(8)$	2	$\mathbf {0.88\,(2)}$	1	3.0
	*Local Path Index*	0.69	7	$0.69\,(9)$	5	$0.69\,(6)$	2	$\mathbf {0.88\,(2)}$	1	3.8
Local	*Resource Allocating Index*	$\mathbf {0.71}$	1	$0.63\,(8)$	6	$0.58\,(3)$	6	$0.78\,(2)$	5	4.5
	*Adar-Academic Index*	$\mathbf {0.71}$	1	$0.63\,(8)$	6	$0.58\,(3)$	6	$0.78\,(2)$	5	4.5
	*Common Neighbour*	$\mathbf {0.71}$	1	$0.63\,(8)$	6	$0.58\,(3)$	6	$0.77\,(2)$	8	5.3
	*Jaccard Coefficient*	$\mathbf {0.71}$	1	$0.62\,(8)$	9	$0.58\,(2)$	6	$0.77\,(2)$	8	6.0
	*Preferential Attachment*	0.63	10	$0.70\,(7)$	4	$0.69\,(6)$	2	$0.78\,(1)$	5	5.3
	*Local Leicht-Holme-Newman*	0.70	6	$0.62\,(7)$	9	$0.57\,(2)$	10	$0.77\,(2)$	8	8.3
	*Baseline*	0.5	11	0.5	11	0.5	11	0.5	11	11.0

The AUC scores for each network are in the left-hand column, the rank of the score in the right-hand column. The errors quoted on the AUC scores are standard deviations from all the computed network snapshots of the selected time scale except for Turkish Shareholder networks where only two predictions are made. We highlight the highest result and any whose result is within the error quoted on the largest result. The baseline method for AUC is the random clustering of node pairs into two groups, and the AUC is 0.5 which means it is like ‘tossing a coin’. The Type column indicates if the link prediction method probes only ultra scales (path length two) or global scales (infinte path lengths).

**Figure 8:**
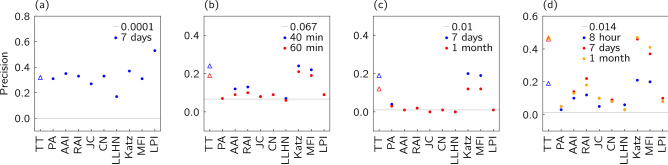
Precision scores for the link prediction results for ten algorithms applied to four temporal networks constructed from real data sets: (**a**) Turkish Shareholder networks, (**b**) Hypertext networks, (**c**) College Message networks, (**d**) Email networks. Results for the Triad Transition algorithm based on transition matrix denoted by the triangle symbol $\bigtriangleup$. For the networks in (**b**–**d**), we also show the results for different time scales (window). See Table 2 for abbreviations used in indicate link prediction methods.

Figure 8 shows that there are some clear patterns in our results for precision. First, each link prediction method has a similar performance relative to the other methods on three of the networks: the Hypertext network (b), the College Message network (c), and the Email network (d). On these three data sets, three algorithms are consistently better than the others though with similar performances relative to one another: our Triplet Transition (TT) method, the Katz method^60,61,66^, and the Matrix Forest Index^64^ (MFI) method. All of these are probing non-local information in the networks, suggesting this is necessary to understand the time evolution of these networks.

**Table 5:** Summary of the average precision scores (on the left) and their ranks (on the right) for selected $\Delta t$ based on k-means clustering.

Type	Algorithm	Shareholder		Hypertext		CollegeMsg		Email		Avg.
	(Time scale)	(2 years)		(1h)		(1 mon)		(1 mon)		Rank
	$[\overline{f_\mathscr {E}(s)}]$	[0.63]		[0.52]		[0.93]		[0.50]		
Global	*Triplet Transition*	0.27	5	$0.19\,(9)$	2	$0.11\,(4)$	2	$\mathbf {0.47\,(6)}$	1	2.5
	*Katz* ($\beta =0.01$)	0.25	8	$\mathbf {0.22\,(8)}$	1	$0.11\,(4)$	2	$\mathbf {0.47\,(6)}$	1	3.0
	*Matrix Forest Index*	0.19	9	$0.19\,(9)$	2	$\mathbf {0.12\,(3)}$	1	$0.4\,(5)$	3	3.8
	*Local Path Index*	$\mathbf {0.36}$	1	$0.09\,(4)$	5	$0.009\,(2)$	7	$0.08\,(3)$	7	5.0
Local	*Resource Allocating Index*	0.28	4	$0.10\,(6)$	4	$0.019\,(4)$	5.0	$0.18\,(7)$	4	4.3
	*Adar-Academic Index*	0.33	2	$0.09\,(5)$	5	$0.008\,(2)$	8	$0.13\,(5)$	5	5.0
	*Common Neighbour*	0.31	3	$0.09\,(5)$	5	$0.008\,(2)$	8	$0.08\,(1)$	7	5.8
	*Jaccard Coefficient*	0.25	7	$0.08\,(6)$	8	$0.0010\,(9)$	10	$0.10\,(4)$	6.0	7.8
	*Preferential Attachment*	0.27	6	$0.07\,(6)$	9	$0.03\,(1)$	4	$0.059\,(6)$	9	7.0
	*Local Leicht-Holme-Newman*	0.09	10	$0.06\,(5)$	11	$0.0001\,(2)$	11	$0.03\,(1)$	10	10.5
	*Baseline*	0.0001	11	0.067	10	0.010	6	0.014	11	9.5

The errors quoted are standard deviations from all the computed network snapshots of the selected time scale, except for theTurkish Shareholder networks which have one prediction for three snapshots.

Overall we see some similarity in these AUC results, summarised in Table 4 as we saw for precision in Table 5. The same three algorithms, the Triplet Transition (TT) methods, the Katz method, and the MFI method, have a similar high performance on the same three networks, the Hypertext network, the College Message network and the Email network. For these three networks, we might also pick out the Preferential Attachment (PA) algorithm. However, now the AUC values for the Turkish Shareholder network show that our Triplet Transition method continues to perform well, unlike for the precision measurements. The Katz, Matrix Forest Index and Preferential Attachment methods are, though, in a weaker group of algorithms as measured by their AUC performance on theTurkish Shareholder network.

For three of these networks, we also measure AUC over snapshots of these networks defined over different time scales. The comparison among methods rarely changes. However, the performance of most algorithms is altered by the size of the time window chosen in some cases, reflecting inherent timescales in the different systems. The most noticeable is that for the Email network, there is a large difference between windows of eight hours and one week but not so much change between a week and one month. The gaps may suggest that if a person is going to produce an email, perhaps following up an email request of bringing a third person into the conversation, that new email is done often on the scale of a few days not always on the scale of a few hours.

The Triplet Transition proposed here is used to predict a link between two nodes by considering these alongside a third node which can be in any position in the network. In this way, this third node not only captures the higher-order interactions but also enables the method to encode both local and non-local information about the link of interest. We find that our Triplet Transition method and the two other global methods used here, Katz Index method^60,61,66^ and the Matrix Forest Index^64^ are generally the best for most of networks we studied, in particular for the Hypertext networks, the College Message networks and the Email networks. As the most successful methods here perform better than other approaches based on local measures, this shows that in most systems the pattern of connections depends on the broader structure of interactions. This dependence of the behaviour of systems on structure beyond nearest neighbours is the crucial motivation for using the language of networks rather than just looking at the statistics of pairs^73^. For instance, the Katz method counts the number of paths between each pair of nodes, probing all paths though giving less weight to longer paths, so nearest neighbours contribute the most.

The results for theTurkish Shareholder networks were a little different. In this case, predictions based on local measurements (paths of length two), the semi-local Local Path Index method (LPI), as well as our non-local triplet transition method outperformed the other global methods in terms of AUC, see Table 4. The global methods also perform poorly on precision, see Table 5. An algorithm with low precision and high AUC, such as Jaccard Coefficient (JC), is predicting the disconnected pairs well.

## Discussion

In this paper, we considered the temporal evolution of networks by looking at a sequence of snapshots of each network. The network $\mathscr {G}(s)$ defined for snapshot *s* contains all the links present between nodes for a certain period, $\Delta t$. Each snapshot *s* covers the period $\Delta t$ immediately following the latest time included in the previous snapshot. In some cases, we have looked at the effect of changing the size of the temporal windows $\Delta t$ on our results. The main tool we use to study the network evolution is the transition matrix $\mathsf {T}$ of Eq. (2) which are derived from the evolution of three-node combinations in a network from one temporal snapshot $\mathscr {G}(s-1)$ to the next $\mathscr {G}(s)$. These transition matrices are obtained from real data by counting the different states of three nodes found in consecutive network snapshots.

To decode the higher-order interaction patterns of network dynamics, we fitted a pairwise interaction model to the real data and computed the transition matrices from the fitted parameters. By comparing the actual transition matrices found numerically with those predicted using a simple pairwise model, as shown in Fig. 3, we demonstrated that higher-order interactions are needed to understand the evolution of networks. For example, in the Email network, derived from the emails within an EU Institution, if three nodes are connected in the previous temporal snapshot, they are less likely to be disconnected in the following snapshot.

To show that we must look beyond the interactions of neighbours, we then designed a link prediction algorithm based on the transition matrix $\mathsf {T}$ of Eq. (2), our Triplet Transition (TT) method. We compared this method with nine other link prediction methods as well as to simple baseline measures. What we found was that on a range of different temporal networks, the Triplet Transition method was as good as two methods based on non-local (global) information in the network, namely, the Katz Index method^60,61,66^ and the Matrix Forest Index (MFI) method^64^. While not always the best on every network or every measure, these three global methods were usually better than the other methods we studied, all of which used information on paths of length two in the network. Intriguingly, the one other method that used paths of length three, Local Path Index^61,63^ (LPI), often performed well too though rarely as well as the top three global methods.

Since the most successful methods in our tests were those that access non-local information, it seems that such information is essential in the evolution of most networks and therefore, it is important to include this in network measures. However, including information from the whole network is numerically intensive and, for any reasonably sized network, the evolution of a link is unlikely to depend directly on what is happening a long way from that link. One reason why our Transition Triplet method works well is that it does not emphasise the vast majority of the network. Most of the information in the transition matrix network is based on neighbours of one or other of the link of interest. For large networks, we use sampling to add the necessary global information into the transition matrices. The Katz index method does include information from all scales but suppresses contributions from more distant parts of the network. The success of the transition triplet approach suggests that there is no need to access all of the global information of a network in order to know what is going on locally. Notably, the overall better performance of the Triplet Transition method reveals that the information of different higher-order interaction patterns can help understand and predict different dynamics of networks.

There is also another reason why our Triplet Transition method may work better than the local methods we look at, and that is because our method is also probing a longer time scale as well as a longer spatial scale. That is we use *two* snapshots, $\mathscr {G}(s-1)$ and $\mathscr {G}(s)$ in order to create the transition matrix $\mathsf {T}$ which in turn we use for the predictions in snapshot $\mathscr {G}(s+1)$. All the other methods used here are based on information from one snapshot $\mathscr {G}(s)$. So again, the success of the Triplet Transition method points to correlations over short but non-trivial time scales as being important in understanding network evolution. In our case, the dependence of results on the time intervals can be seen in the effect of $\Delta t$ used to define the transition matrices are important. For different data sets, we found different $\Delta t$ gave optimal results which of course reflects inherently different timescales in the processes encoded by our different data sets. So another conclusion is that higher order effects in terms of both time and space are needed to understand the evolution of temporal networks and to make effective predictions for links. The inclusion of higher-order order effects in terms of time remains undeveloped relative to the work on higher-order spatial network features.

In our approach we have focused on changes in the edges and ignored changes in the set of nodes. Our method can include nodes which are not connected in some or even all our snapshots provided these nodes are known. However, in many cases the data sets may only record interactions, our edges, and our set of nodes is only inferred from that. As a result we often have no information about such (totally isolated) nodes. Suppose we are given a list of emails, our edges, sent at a given time between email accounts, our nodes. We cannot distinguish accounts which are dormant from those that are deleted or added to the system without additional information. Should we have such data on node birth and death, then in some cases it might be an important process to include, but it is not one we address in our approach.

Currently constructing optimal higher order models is a timely topic^5^. Our method can be generalised to include even higher order interactions, such as quadruplet and so on. The method can be used to indicate higher order evolutionary mechanisms in a network and suggest what is the most likely order of interaction. Our work shows that studying the evolution of small graphs over short time periods can reveal important information and predictions regarding network evolution.

## Supplementary Information

Supplementary Information.
