# Supplementary material for: Higher-order temporal network effects through triplet evolution
Source: Sci Rep. 2021 Jul 29;11:15419. doi: 10.1038/s41598-021-94389-w (PMC8322211; doi:10.1038/s41598-021-94389-w)
Supplement: Supplementary file 1 — Supplementary Information. [file 41598_2021_94389_MOESM1_ESM.pdf]

Supplementary Information
Appendix for
Higher-Order Temporal Network Effects through Triplet Evolution

Qing Yao[1,2], Bingsheng Chen[1,2], Tim S. Evans[1,3], Kim Christensen[1,2],

18th May 2021

(1) Centre for Complexity Science, Imperial College London, London, SW7 2AZ, U.K.
(2) Condensed Matter Theory Group, Imperial College London, London, SW7 2AZ, U.K.
(3) Theoretical Physics Group, Imperial College London, London, SW7 2AZ, U.K.

A Transition matrix estimation

The memory needed for the calculations in our Triplet Transition (TT) method scales with the number of combination of triplet in network, that is $\binom{N}{3} = N(N-1)(N-2)/6 \sim O(N^3)$ and this can be seen in Figure A1.

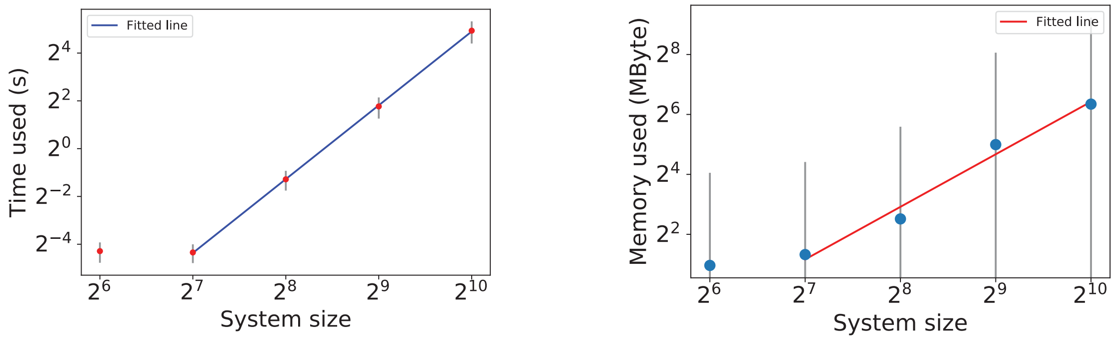

Figure A1: Time and memory needed for generating all three node graphlet combinations against a different number of nodes. $N = 2^n$ $n = 6, 7, 8, 9, 10$. For each n, the time and memory for each run are measured and the error bars are corresponding to standard deviation. The time used scales as expected order (the slope of the fitted line is 3.09 ± 0.02) of the system size. The slope of the fitted line for the memory used is 1.75 ± 0.02.

To ensure the number of triplets is sufficiently large enough to estimate the transition matrix $\widehat{\mathsf{T}}$, we use all three-node combinations if the number of nodes in the system is smaller than 10^3; otherwise, we sample 10^5 triplets chosen uniformly at random from the set of all three-node combinations. If the calculated $\widehat{\mathsf{T}}$ is stable, we choose this sample number for the following analysis. Otherwise, we continue to add sample a further sample of 10^5 triplets until the $\widehat{\mathsf{T}}$ is stable.

B Simple Null Models

The simplest null model for the evolution of the network is one in which we assign a probability p that in each time step, a pair of nodes changes from disconnected to connect with probability p. In contrast, a connected pair becomes disconnected with probability q. We can then write down the transition matrix in this Pairwise Null model $\mathsf{T}^{(\mathrm{pw})}(p, q)$ (often abbreviated to $\mathsf{T}^{(\mathrm{pw})}$) for our three-node combinations in terms of the set of four states $\mathcal{M}^4 = \{m_0, m_1, m_2, m_3\}$ where

m_i is the configuration in which three nodes have i links between them. Namely

$$\mathsf{T}^{(\mathrm{pw})}(p,q) = \begin{pmatrix} (1-p)^3 & 3p(1-p)^2 & 3p^2(1-p) & p^3 \\ q(1-p)^2 & (1-q)(1-p)^2 + 2qp(1-p) & 2(1-q)p(1-p) + qp^2 & (1-q)p^2 \\ q^2(1-p) & 2(1-q)q(1-p) + q^2 p & (1-q)^2(1-p) + 2qp(1-q) & (1-q)^2 p \\ q^3 & 3(1-q)q^2 & 3q(1-q)^2 & (1-q)^3 \end{pmatrix} . \tag{B1}$$

Here $\mathsf{T}^{(\mathrm{pw})}_{ij}$ is the probability that three nodes connected with i nodes, in state m_i, evolves in the next time step to a configuration m_j with j edges between those three nodes. For instance, $\mathsf{T}^{(\mathrm{pw})}_{03}$ denotes the probability of evolving from three disconnected nodes m_0 to state m_3 of three fully connected nodes. The addition of an edge for each of the three edges gives a single factor of p, so $\mathsf{T}^{(\mathrm{pw})}_{03} = p^3$ overall. When looking at the transition from a single edge to two edges in a triplet, the entry $\mathsf{T}^{(\mathrm{pw})}_{12}$, we can do this in two ways. First, we can add one new edge with probability p, keeping the other pair of unconnected nodes in that state, probability (1-p), and keeping the original single connected pair in that state with probability $(1-q)$. There are two ways of choosing where to add the extra edge, so we have a factor of $2p(1-p)(1-q)$. However, we could also remove the existing edge and add two new edges between the other pairs of edges edge in one of two positions, giving the second factor of $p^2 q$ seen in the entry for $\mathsf{T}^{(\mathrm{pw})}_{12}$ in (B1). We can check that the rows sum to one, $1 = \sum_j \mathsf{T}^{(\mathrm{pw})}_{ij}$ since we conserve the total number of triplets in our models.

Our second null model, our "edge swap model", we swap the ends of a pair of edges so edges (u,v) and (w,x) in snapshot s are removed and are replaced by edges (u,x) and (w,v) in the next snapshot. This preserves the degree of every node. For each update from $\mathcal{G}(s)$ to $\mathcal{G}(s+1)$ we update 20% edges.

The final model, our "random walk model", is also an edge rewiring model but it is based on higher-order structures as we use random walks to select the new edges. The model starts with an Erdős-Rényi graph. The initial node for a random walker, say u, is chosen uniformly at random from the set of nodes. Then one of the edges from u, say the edge to a node y is chosen uniformly from the set of neighbours. Finally, three non-backtracking steps are made on the network starting from u and ending at a node x, in which edges are always chosen uniformly from those available excluding any edge used in the previous step of the random walk. The existing edge (u,y) is removed and replaced by a new edge (u,x). The final graph is then created through a projection where nodes are connected if they share a common neighbour, that is if directed edges from u to v and w to v exists, then the projected graph has an undirected link between u and v. This rewiring and projection procedure maintains the number of edges and nodes in the original graph but not in the projected graph.

To produce the time evolution, we rewire 20% of the edges using this rewiring procedure and then use this new network as the next snapshot. In our context, our random walk model is used to produce test networks with local correlations between nodes. While motivated by real-world examples and building on existing experience with the model [1], here it is used as a toy model to illustrate the approach.

We can use these three simple models to generate artificial temporal networks to test our approach. The results in terms of the transition matrix $\widehat{\mathsf{T}}$ derived from the artificial networks are shown in Figure B2. To show the deviation from the our pairwise interaction null model, we look at the difference $\Delta\widehat{\mathsf{T}}(s)$ between actual results for the average of $\widehat{\mathsf{T}}(s)$ and those predicted in the null model $\widehat{\mathsf{T}}^{(\mathrm{pw})}(s)$, so

$$\Delta\widehat{\mathsf{T}}(s) = \widehat{\mathsf{T}}(s) - \widehat{\mathsf{T}}^{(\mathrm{pw})}(s) . \tag{B2}$$

The behaviour of our simple "pairwise model" should be completely captured by the reference transition matrix $\widehat{\mathsf{T}}^{(\mathrm{pw})}(s)$ and, as expected, the numerical results shown in Figure B2a show no significant difference between numerical data $\widehat{\mathsf{T}}^{(\mathrm{pw})}(s)$ and theoretical $\mathsf{T}^{(\mathrm{pw})}$.

For Figure B2b, we use the artificial networks generated by the "edge swap model". While this involves two pairs of edges, so in principle is a higher-order model, in a sparse graph, the four nodes selected by a pair of randomly selected edges are unlikely to be linked by other edges. So in practice, in terms of the triplet graphlets, this model behaves much like the pairwise model and shows little difference from that model.

It is only with the networks generated using our three-step random walk that we see significant differences between the data and the pairwise model. This is to be expected as higher-order processes were used to create the numerical networks.

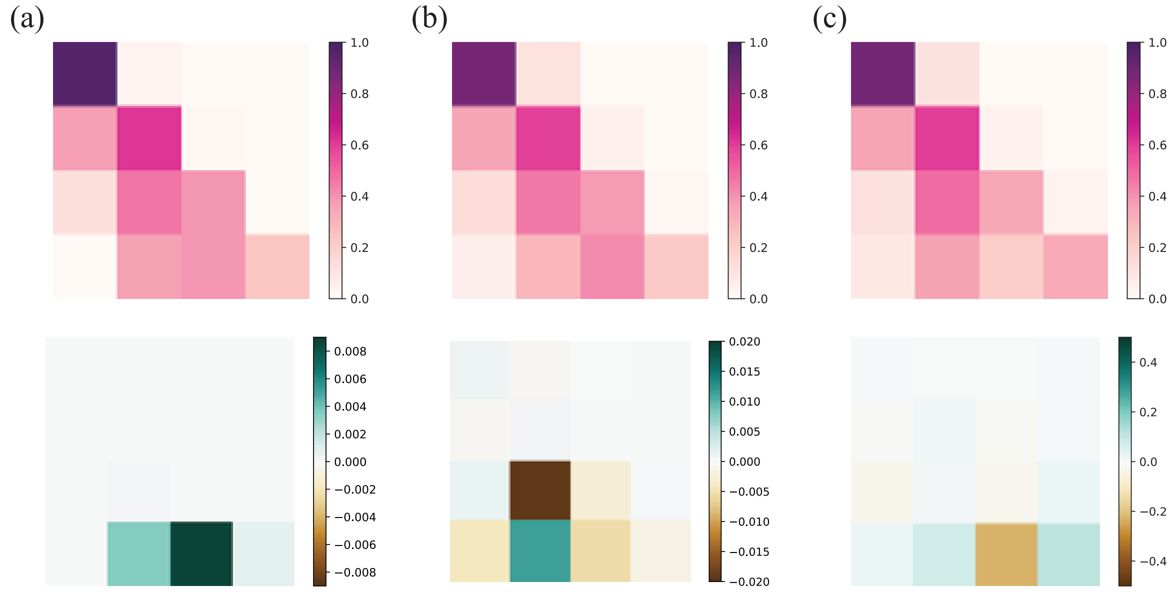

Figure B2: The triplet transition matrix $\widehat{\mathsf{T}}$ estimated from the artificial data. Each of the six four-by-four heat maps shows values where the rows represent one initial triplet graphlet i and the columns representing the final graphlet j using our unlabelled graphlet $\mathcal{M}_4$ set of triplets to make the visualisations manageable. The top three heat maps show the average value of entries $\langle \widehat{\mathsf{T}}_{ij} \rangle$. The bottom row of three heat maps give the values of the difference matrix $\langle \Delta\widehat{\mathsf{T}} \rangle = \widehat{\mathsf{T}}^{(\mathrm{pw})} - \widehat{\mathsf{T}}$ of (B2) in which the numerical data is compared to the analytical form predicted from the simple pairwise model of (B1). The scales for the colours of the first two heat maps on the bottom row are much smaller (a factor of twenty or more) than for the results for $\langle \Delta\widehat{\mathsf{T}} \rangle = \langle \widehat{\mathsf{T}}^{(\mathrm{pw})} \rangle - \langle \widehat{\mathsf{T}} \rangle$ in the random walk model shown in the bottom right corner. Any large entries in these lower rows of heatmaps indicate higher-order effects not present in our simple pairwise model of $\widehat{\mathsf{T}}^{(\mathrm{pw})}$. The three columns of heat maps show results for the three different artificial temporal networks created numerically using the stochastic models defined in Section B: (a) our "pairwise model", (b) our "edge swap model", and (c) our "random walk model". Only the networks formed with random walks show significant higher-order effects.

C Data Sets

In this project, we use several different datasets to produce temporal networks with different resolutions. The resolution of a network is the time interval used to create each snapshot $\mathcal{G}(s)$ of our network. This can be done in two ways, depending on the context. In either case, the resolution can be 'seconds', 'hours', 'days' or 'months' and should be chosen to suit the context.

In the first type of temporal data, the data capture interactions between pairs of nodes which occur briefly on the time scale of the network resolution. A list of the times of phone

calls between members of a social network would be an example of this type of data. In this case, each edge in a single snapshot indicates that an event linking the two nodes occurred during the time interval.

The second approach is where the pairwise interactions recorded in the data typically last for much longer than the resolution, but they do change slowly over time. An example of this would be hyperlinks between webpages. In this second case, the snapshots are the network at one instant in time, and the interval is now the time between these snapshots.

We use five different data sets which are as follows.

- `Turkish Shareholder Network` (`Shareholder`).

  The nodes are shareholders in Turkish companies. The shareholders are linked if they both hold shares in the same company during the time interval associated with the snapshot [1].

- `Wikipedia Mathematician` (`WikiMath`). Each biographical Wikipedia page of an individual mathematicians forms a node. If a hyperlink links two biographies (in either or both directions), a link is present in the network. The edges are edited by users and are both added and removed over time. Each snapshot represents the state of these webpages at one moment in time. The data is taken at one point in three different years, 2013,2017, and 2018, so the intervals are not constant in this case. See the paper [2] for further details on this dataset.

- `College Message` (`CollegeMsg`). The nodes are students, and an edge in a snapshot indicates that the students exchanged a message within the interval associated with that snapshot. The data was collected over a seven month period in 2004, see [3, 4, 5] for more details.

- `Email` (`Email`). This is derived from the emails at a large European research institution sent between October 2003 and May 2005 (18 months). Each node corresponds to an email address. An edge in a given snapshot indicates that an email was sent between the nodes in the time interval corresponding to that snapshot [5, 6, 7].

- `Hypertext` (`Hypertext`). This is the network of face-to-face contacts of the attendees of the Association of Computing Machinery (ACM) Hypertext 2009 conference. In the network, a node represents a conference visitor, and an edge represents a face-to-face contact that was active for at least 20 seconds [8, 9].

We provide a summary of the graph statistics in Table C1. Some further information on the temporal characteristics of some of the data sets is given in Figures C5 – C9.

Dataset	(Abbreviation)	Nodes (N)	Edges (E)	Time Period (T)	Resolutions (Δt)	Source
Turkish Shareholder	(`Shareholder`)	39901	68017	2010,2012,2014,2016	2 years	[1]
Wikipedia Mathematician	(`WikiMath`)	6049	36315	2013,2017,2018	1 and 4 years	[2]
College Message	(`CollegeMsg`)	1899	20296	6 months, 2004	7 days, 1 month	[3, 4, 5]
Institution Email	(`Email`)	986	24929	1 year, 1970-1971	8 hours, 7 days, 1 month	[5, 6, 7]
Hypertext	(`Hypertext`)	113	5246	3 days in 2009	40,60 min	[8, 9]

Table C1: The detailed information of graph statistics.

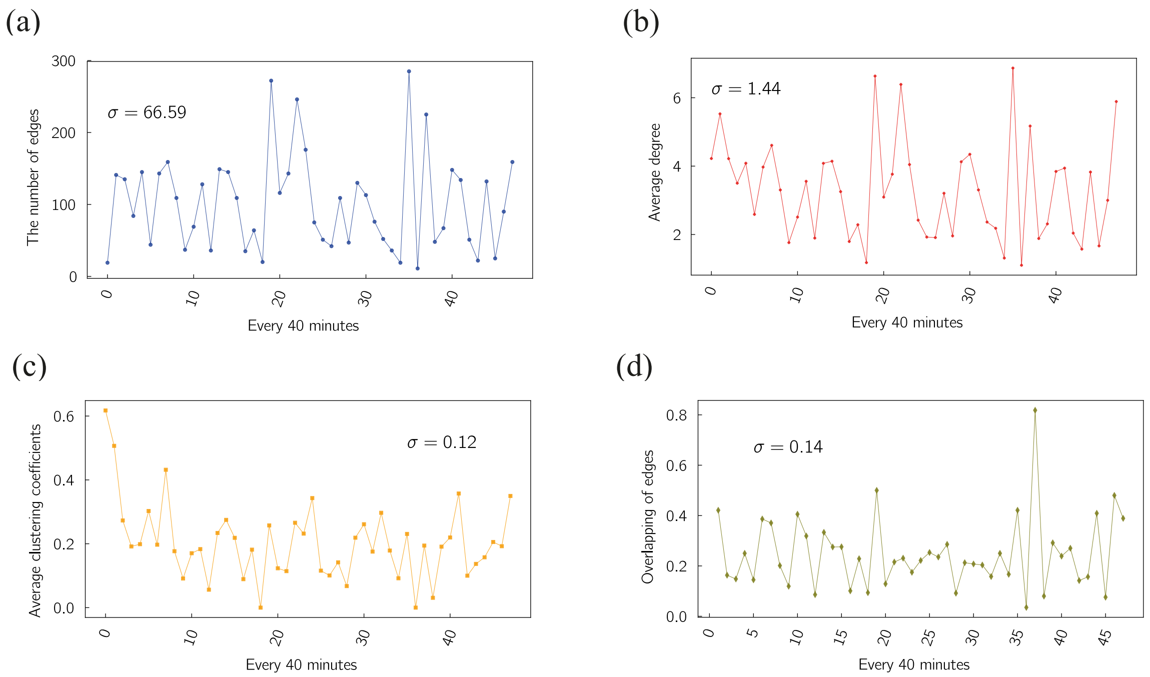

Figure C3: Temporal evolution of graph statistics and their standard deviation for Hypertext network every 60 minutes from June 2009 till July 2009. There are 48 graphs in total.

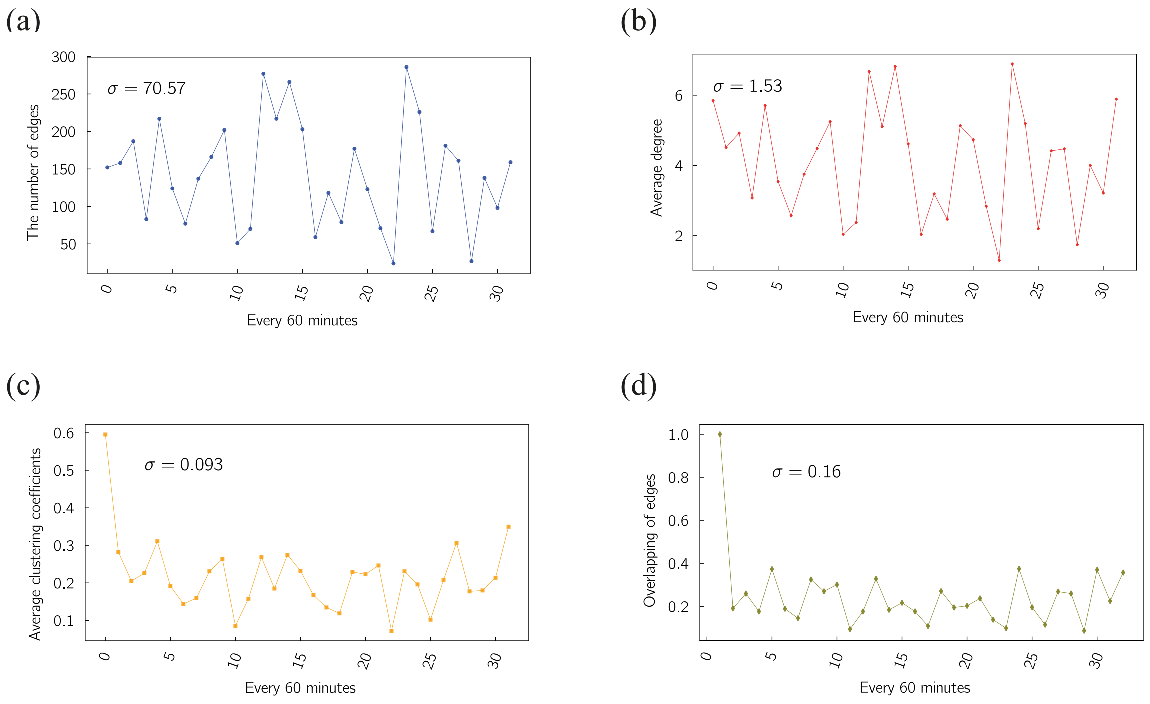

Figure C4: Temporal evolution of graph statistics and their standard deviation for Hypertext network every 40 minutes from June 2009 till July 2009. There are 32 graphs in total.

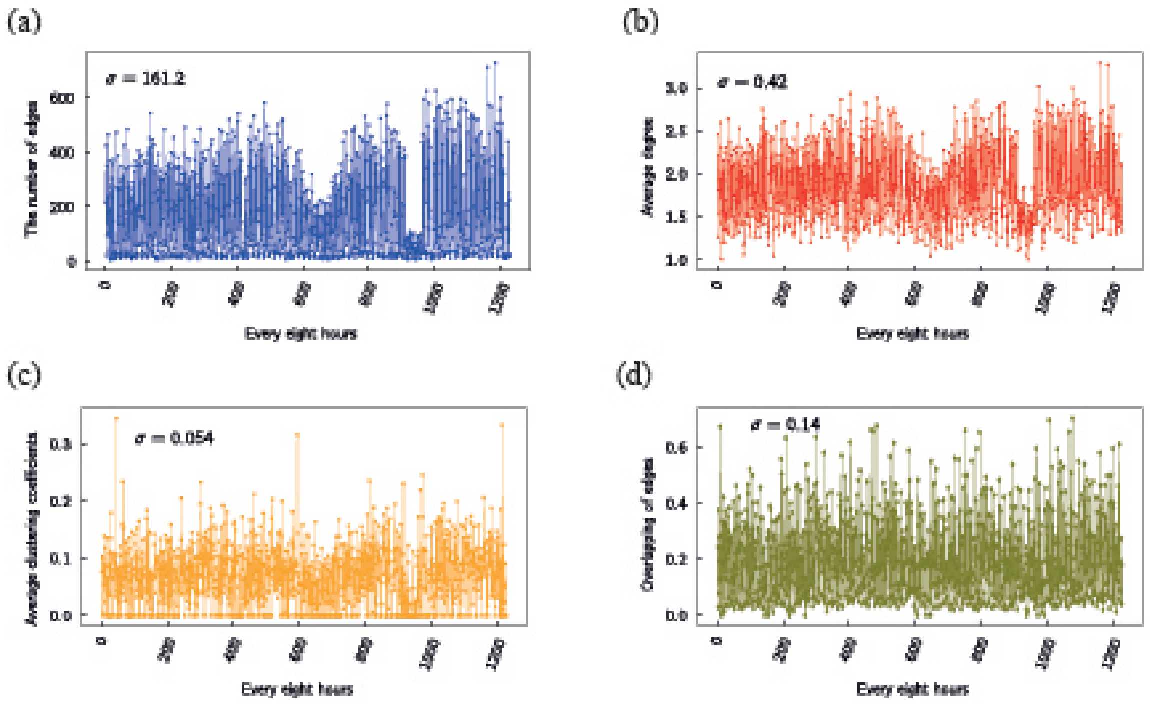

Figure C5: Temporal evolution of graph statistics and their standard deviation for Email network every eight hours from Jan 1970 till March 1972.

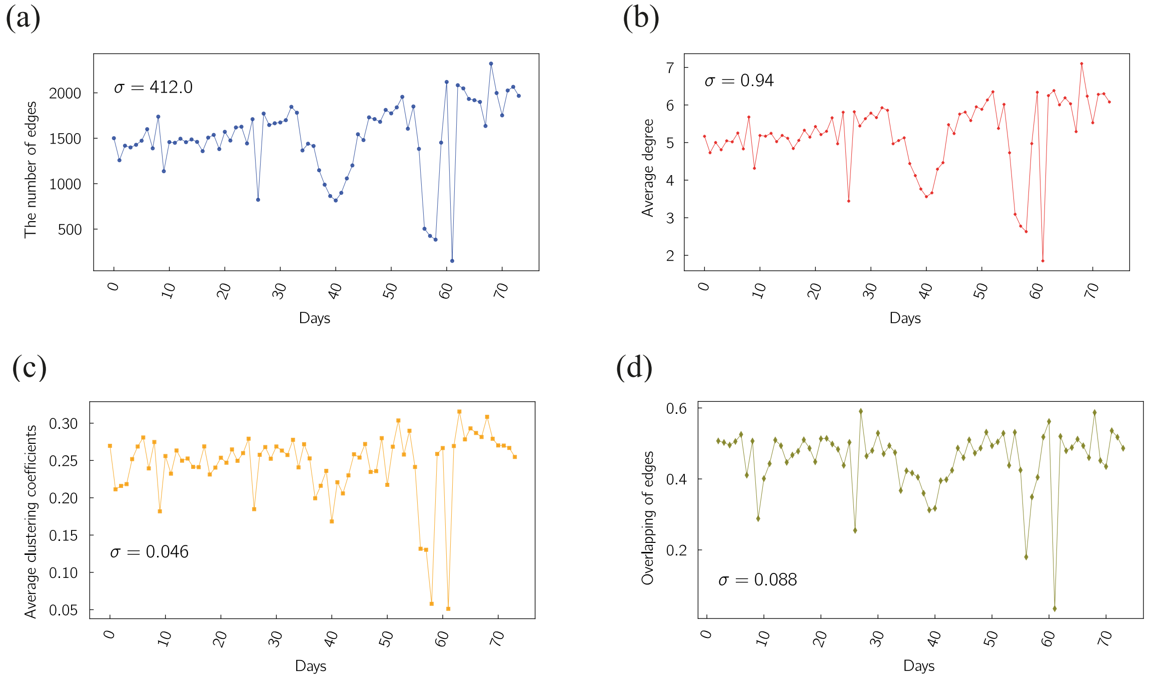

Figure C6: Temporal evolution of graph statistics and their standard deviation for Email Networks every seven days from Jan 1970 till March 1972.

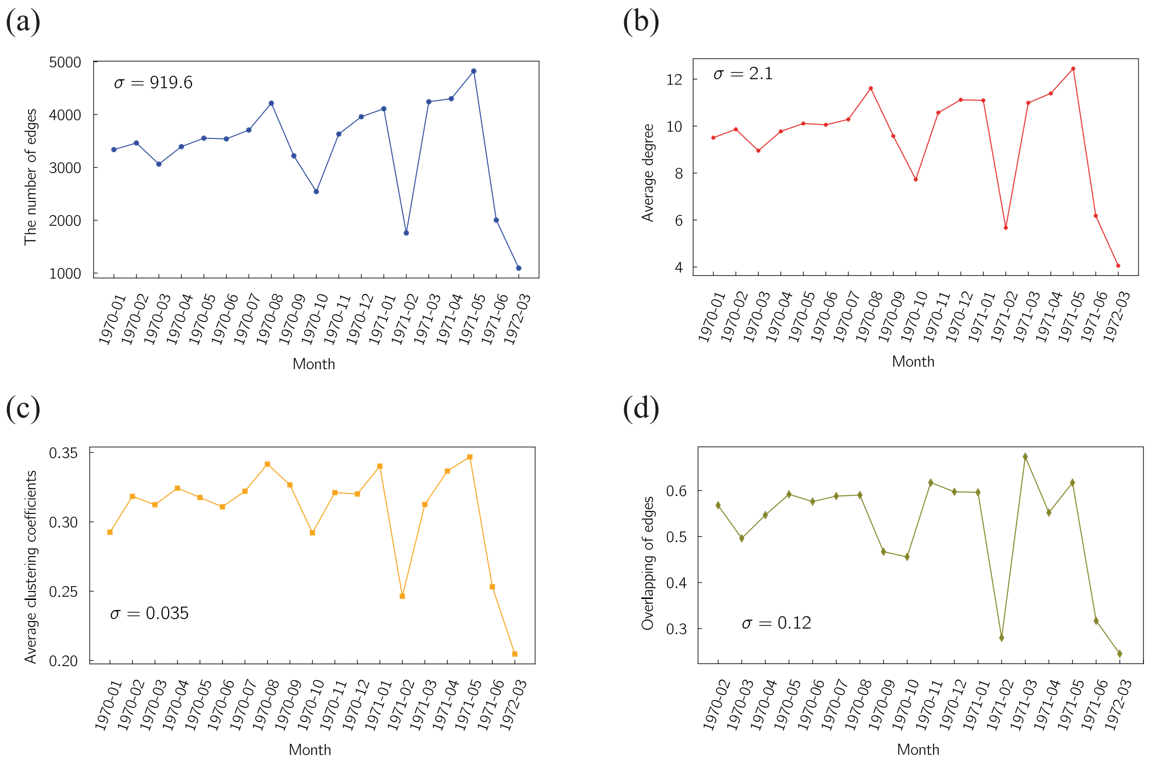

Figure C7: Temporal evolution of graph statistics and their standard deviation for Email network every one month from Jan 1970 till March 1972. There are 19 snapshots in total and the overlapping of edges starts from Feb 1970 that is the overlapping between Jan 1970 and Feb 1970.

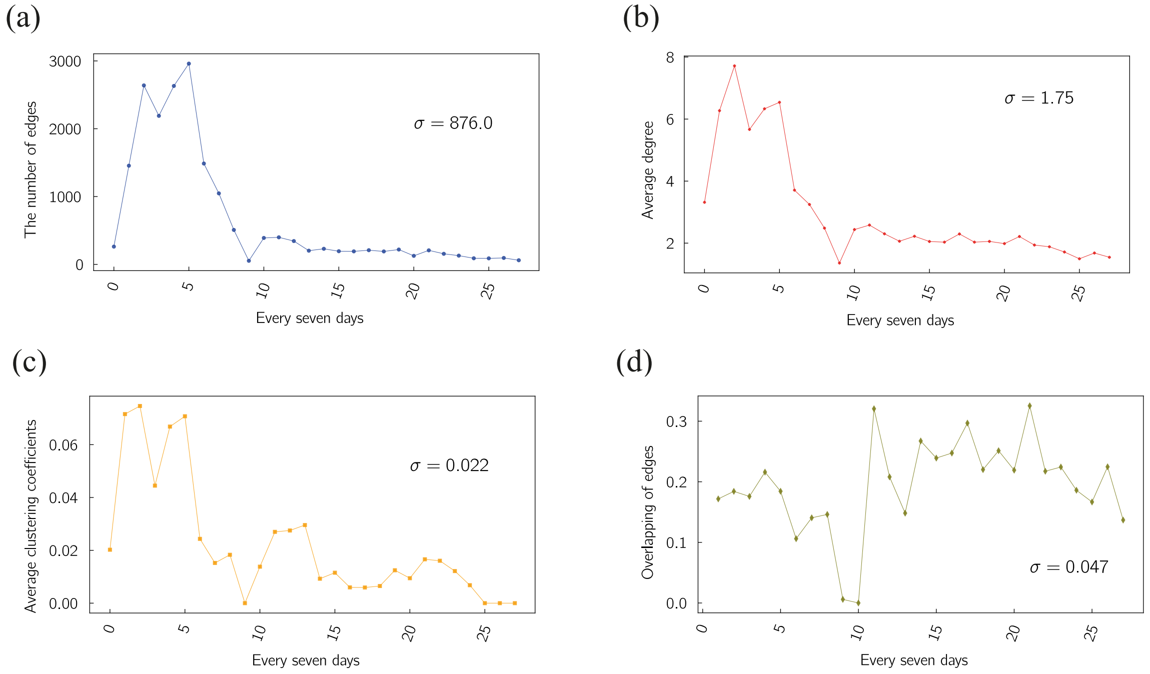

Figure C8: Temporal evolution of graph statistics and their standard deviation for College Message every 7 days from April 2004 till Oct 2004. There are 28 snapshots in total.

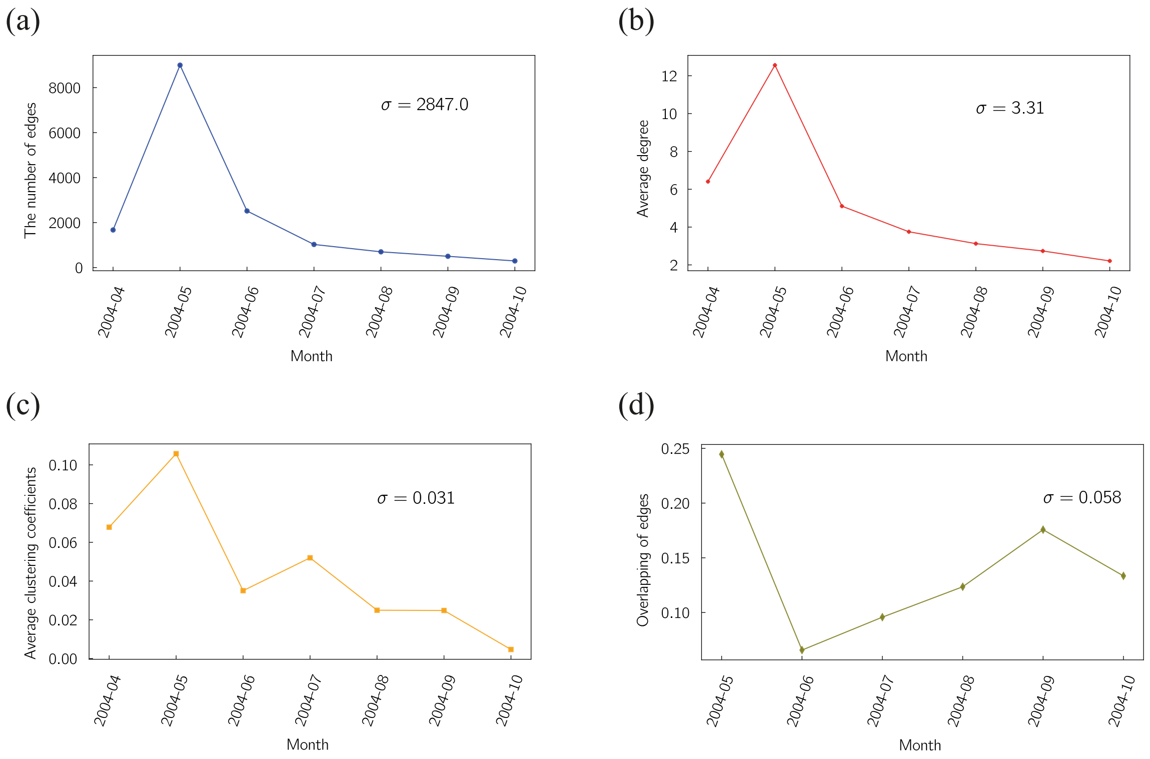

Figure C9: Temporal evolution of graph statistics and their standard deviation for College Message every month from April 2004 till Oct 2004. There are 7 snapshots in total and the overlapping of edges starts from May 2004 that is the overlapping between April 2004 and May 2004.

D Significance test of the pairwise interactions

The significance of the transition of the triplet transition can be quantified by using the z-score (standard score). The z-score has been used to a qualitative measure of statistical significance of different motifs [10] or temporal motifs [11]. We apply similar procedures to compute z-scores for different triplet transitions and for a transition from graphlet m_i to graphlet m_j we define

$$Z_{ij} = \frac{\langle \widehat{\mathsf{T}}_{ij}(s) \rangle - \langle \widehat{\mathsf{T}}^{(\mathrm{pw})}_{ij}(s) \rangle}{\sigma^{(\mathrm{pw})}_{ij}} \, . \tag{D3}$$

Here $\sigma^{(\mathrm{pw})}_{ij}$ is the standard deviation in the ij-th entry of the transition matrices $\widehat{\mathsf{T}}^{(\mathrm{pw})}(s)$ obtained from the simple pairwise model.

To calculate this, we generate an ensemble of $R = 1000$ realisations of the null model, the pairwise null model. The number of simulations R needed was found as follows. We start from $R = 100$ realisations, increase to 200, 300 and so on. Each time we increase R we compare the difference in the results to those found with the $(R - 100)$ realisations; if the results of the transition counting do not change from $(R - 100)$ to R, we assume R is sufficient, and we stop increasing R. Otherwise, we continue to increase the number of realisations until the results do not change. The values of p and q used in the calculation of $\widehat{\mathsf{T}}^{(\mathrm{pw})}_{ij}(s)$ are those inferred from one real network as described in (6) and (5).

A z-score Z_{ij} with absolute value much bigger than one shows us that the data has behaviour not accounted for in our simple pairwise model. So it quantifies how likely a specific transition from state i to j is derived from higher-order processes not captured by simple pairwise interactions. The results for Z_{ij} for some of our networks are shown in Figure D10.

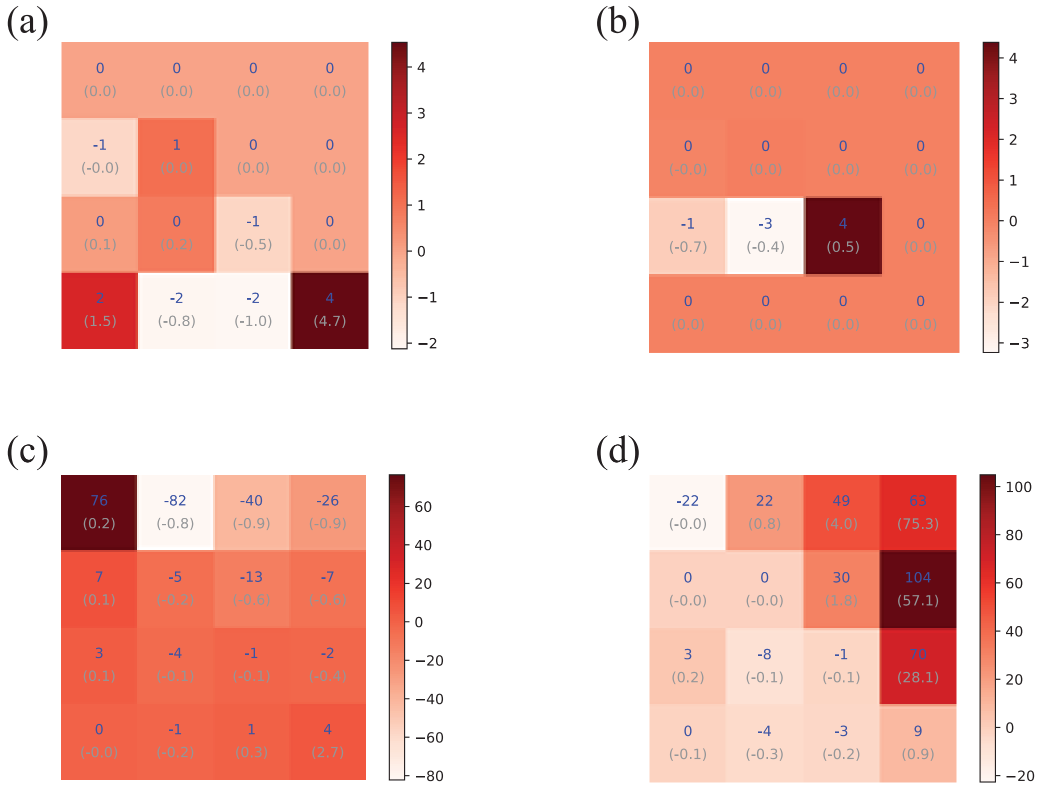

Figure D10: Z-scores for triplet transition based on comparisons of data to the pairwise simple model. Each of the large four squares corresponds to a Z matrix (D3) for a different data source, labelled as follows: (a) Turkish Shareholder network ($\Delta t = 2$yr), (b) Wikipedia Mathematician ($\Delta t = 1$yr), (c) College Message data ($\Delta t = 1$mo), and (d) Email network data ($\Delta t = 1$mo). Each large square represents a four by four grid of smaller squares. The rows (columns) are the triplets at the earlier (later) arranged in order of size from smallest m_0 to largest m_3 going top to bottom (left to right). In each the colour represents Z_{ij} on the scale given on the right of each large square, the top blue digits represent Z-scores, and the bottom grey digits in brackets gives $(N^{\mathrm{r}}(m) - N^{\mathrm{pw}})/N^{\mathrm{pw}}$ where N^{r} gives the number of triplets in the real data and N^{pw} gives the number predicted in the simple pairwise model for the same sized sample.

E Other Link Prediction Methods

We have used several other measures as summarised in main text and in Table E2. These methods use node similarity measures to make link predictions and we will describe these scores in what follows.

Abbreviation	Method	Reference	Length Scale	Code
AAI	Adar-Academic Index	[12]	2	`nx`
CN	Common Neighbour	[12]	2	`nx`
JC	Jaccard Coefficient	[12]	2	`nx`
Katz	Katz	[13]	∞	`Own`
LLHN	Local Leicht-Holme-Newman	[14]	2	`Own`
LPI	Local Path Index	[15]	3	`Own`
EE	Edge Existence	[Here]	1	-
PA	Preferential Attachment	[12]	2	`nx`
RA	Resource Allocating Index	[15]	2	`nx`
MFI	Matrix Forest Index	[16]	∞	`Own`
TT	Triplet Transition	[Here]	∞	`Own`

Table E2: Table of the link prediction methods used and their abbreviations. The length scale given indicates the longest path length involved in the method or equivalently the largest power of the adjacent matrix involved in the method. Under code `nx` indicates that a NetworkX [17] routine was used, Own indicates the authors' own code was used. The Edge Existence (EE) approach was not investigated numerically but was included for the sake of comparison.

As we described in the main text, the most obvious similarity measure is the Edge Existence (EE) Index. It is defined as:

$$s_{\mathrm{EE}}(u,v) = A_{uv} = \sum_{e\in\mathcal{E}} \delta_{e,(u,v)} \,. \tag{E4}$$

The Common Neighbours (CN) method [12, 13, 18] simply scores the relationship between two nodes based on the number of neighbours they have in common

$$s_{\mathrm{CN}}(u,v) = \sum_{w\in\mathcal{V}} A_{uw}A_{wv} = |\mathcal{N}(u)\cap\mathcal{N}(v)| \,. \tag{E5}$$

This will tend to give large scores if u and/or v have high degrees.

To illustrate this, we can estimate the number of common neighbours in a random graph with degree distribution $p(k)$. Suppose the vertices u and v have degree k_u and k_v respectively. Then this means we are picking out a pair of stubs with probability $k_u k_v/(4E^2)$ if there are E edges in the simple graph. The probability that a pair of stubs are connected to the same nearest neighbour node of degree k is $(1/2)k(k-1)p_{\mathrm{nn}}(k)$ where $p_{\mathrm{nn}}(k) = kp(k)/\langle k\rangle$ is the probability that the nearest neighbour has degree k. This gives us that the number of nearest neighbours in common in a random graph may be estimated to be

$$s_{\mathrm{CN,rnd}}(u,v) \approx \frac{k_u}{2E}\frac{k_v}{2E}\frac{(\langle k^3\rangle - \langle k^2\rangle)}{\langle k\rangle} \propto s_{\mathrm{PA}}(u,v) \,. \tag{E6}$$

One way to take this bias in $s_{\mathrm{CN}}(u,v)$ towards higher degree nodes is to make the score comparison between the two. If we compare by looking at the difference between s_{CN} and its

expected value in the configuration model we end up with

$$s_{\text{CN,diff}}(u,v) = s_{\text{CN}}(u,v) - \beta' s_{\text{CN,rnd}}(u,v) = \left(\sum_{w \in \mathcal{V}} A_{uw} A_{wv} \right) - \beta \frac{k_u}{2E} \frac{k_v}{2E} \tag{E7}$$

where β is just a network-dependent rescaling of the parameter β. This expression is very similar to a term in a Modularity index used for vertex clustering. On the other hand, if we look at the fractional difference, we could use a score

$$s_{\text{CN,frac}}(u,v) = \frac{s_{\text{CN}}(u,v)}{s_{\text{CN,rnd}}(u,v)} \propto \frac{1}{k_u k_v} \sum_{w \in \mathcal{V}} A_{uw} A_{wv} \tag{E8}$$

and we'll see similar forms in other indices below.

The JACCARD COEFFICIENT (JC) method [12, 18] based on the well known similarity measure [20] in which the likelihood that two nodes are linked is equal to the number of neighbours they have in common relative to the total number of unique neighbours.

$$s_{\text{JC}}(u,v) = \frac{|\mathcal{N}(u) \cap \mathcal{N}(v)|}{|\mathcal{N}(u) \cup \mathcal{N}(v)|} \tag{E9}$$

$$= \frac{|\mathcal{N}(u) \cap \mathcal{N}(v)|}{|\mathcal{N}(u) + |\mathcal{N}(v)| - 2A_{uv} - |\mathcal{N}(u) \cap \mathcal{N}(v)|} \tag{E10}$$

$$= \frac{s_{\text{CN}}(u,v)}{k_u + k_v - 2s_{\text{EE}}(u,v) - s_{\text{CN}}(u,v)} \,. \tag{E11}$$

For instance in a random graph we estimate that

$$s_{\text{JC,rnd}}(u,v) \approx \frac{s_{\text{CN,rnd}}(u,v)}{k_u + k_v - 2A_{uv} - s_{\text{rnd}}(u,v)} \tag{E12}$$

which for large $k_1, k_2 \sim O(K)$ gives $s_{\text{JC,rnd}}(u,v) \sim O(K)$ while $s_{\text{CN,rnd}}(u,v) \sim O(K^2)$.

The RESOURCE ALLOCATING INDEX (RAI) method [15] and the ADAMIC-ADAR INDEX (AAI) method [12, 18] are both based on the idea that if two vertices u and v share some 'features' f . For our simple graphs, the most important feature two nodes can share is an edge between them, while the second most important feature is sharing a common neighbour. The Resource Allocating Index method and the Adamic-Adar Index method ignore the presence or presence of a direct connection between vertices u and v (as captured by s_{EE} of (E4)) and the features considered for these two similarity scores are the the common neighbours themselves $w \in \mathcal{N}(u) \cap \mathcal{N}(v)$. One way to picture this is to remember that the adjacency list representation of a network, often used in practice numerically, is where for each node we record the list of neighbours. We can picture this adjacency list of neighbours $\mathcal{N}(u)$ as the 'words' on the 'document' u to connect with text analysis methods. The frequency of this feature simply is the degree $|\mathcal{N}(w)|$ of the neighbouring vertex w as that is the number of times this feature will occur in the adjacency lists of other vertices. The Resource Allocating Index method and the ADAMIC-ADAR INDEX (AAI) method differ in their choice of weighting function $W(w)$ used to weight the features when defining their similarity score.

The KATZ INDEX (Katz) method [12, 13, 18] counts the number of paths between each pair of vertices, where each path of length ℓ contributes a factor of β^ℓ to the score.

$$s_{\text{Katz}}(u,v) = ([\mathsf{I} - \beta \mathsf{A}]^{-1})_{uv} \tag{E13}$$

In a random graph the probability the two stubs from vertices u and v of degree k_u and k_v respectively are connected is simply $k_u k_v/(4E^2)$. Comparing this to the estimate for the

number of common neighbours in a random graph, (E6), we estimate that in a random graph the Katz score is dominated by the existence of an edge if $\beta \ll (\langle k^3 \rangle - \langle k^2 \rangle)/2$. (here $\beta = 0.01$).

The Matrix Forest Index (MFI) method [16] is defined as

$$s_{\text{MFI}}(u, v) = [(\mathsf{I} + \mathsf{L})^{-1}]_{uv} \,, \tag{E14}$$

where $\mathsf{L} = \mathsf{D} - \mathsf{A}$ is the Laplacian. One way to see this forms a suitable similarity measure is to know that if $\mathsf{Q} = (\mathsf{I} + \mathsf{L})^{-1}$, then $D_{ij} = Q_{ii} + Q_{jj} + Q_{ij} + Q_{ji}$ is a metric on the network and hence is a good distance measure. Similarity measures are often related to distance through a function that ensures the similarity measure increases if the distance between two vertices decreases.

A final way to view the Matrix Forest Index method is to consider a discrete-time version of the diffusion process described by a Laplacian. If we have a vector $\mathbf{w}(t)$ whose entries represent the number of particles at every vertex at time t, then we can define a diffusion process where

$$\mathbf{w}(t+1) - \mathbf{w}(t) = \lambda \mathsf{L} \mathbf{w}(t) \,. \tag{E15}$$

In this process, a fraction λ of the particles at each node flow down each edge in each time step (so λk particles leave each node at each step so large degree nodes to lose a larger fraction of every time step)). The Laplacian gives the network flow into a given vertex, and the number of particles is conserved (since $\sum_i L_{ij} + 0$). The matrix $\mathsf{Q} = (\mathsf{I} - \mathsf{L})^{-1}$ used to give the MFI score is therefore

$$\mathbf{w}(t) = [(\mathsf{I} + \mathsf{L})^{-1}] \mathbf{w}(t+1) \,. \tag{E16}$$

That is if we were to demand that at time $t + 1$ we had only had particles at one site u, then Q_{uv} tells us how many of those particles were at vertex v at the previous time step.

E.1 Scores for other link prediction methods from under-sampling

We under-sampled 1000 link pairs and 1000 non-connected pairs to demonstrate that TT can naturally split the score into two clusters[19], which can be used to predict link existence in main text Figure 4. We also compute the performance of other similarity measures, which shows, apart from Katz score, none of the methods show a good separation into two clear clusters in Figure E11.

E.2 From Node Scores to Link Predictions

Once we have a similarity score for a pair of nodes, we have to turn this into a prediction. Generally, the node pairs with a high similarity score in $\mathcal{G}(s)$ will be predicted to have an edge in $\mathcal{G}(s + 1)$, and low scores will lead to a no edge prediction. If we are looking at a link addition problem [13, 21, 22, 23, 12, 24, 25] or, more generally, an uncertain link problem [18], then the score is turned into a prediction for the node pair by using a standard machine learning approach to what is a binary classification problem. For instance, we remove edges (or add an edge between unconnected nodes [18]) using some examples (say 10%) to train the classifier and then the remain node pairs as used to verify the effectiveness of the method. In the simplest method we could imagine ranking our node pairs based on similarity scores, and then the n_1 unconnected node pairs with the highest scores are assigned an edge, while the n_0 lowest-scoring node pairs which are connected are predicted to have their edges removed. The values for n_0 and n_1 could be learnt as part of the training of this simple classifier. However, more sophisticated methods are normally used.

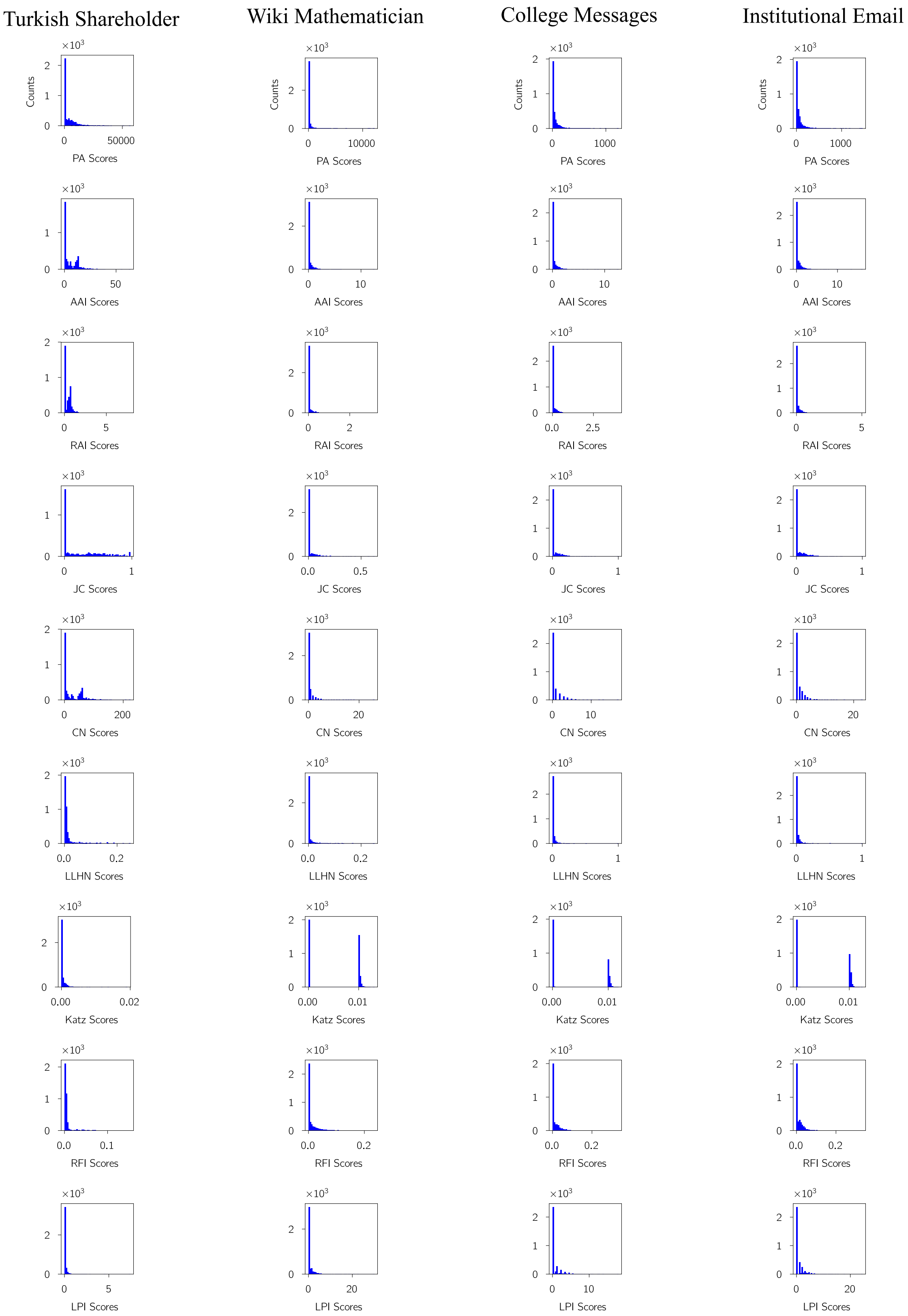

Figure E11: Histograms of similarity scores for nine different link prediction methods applied to four different real world data sets. Each column is for a different data set, from left to right: Turkish Shareholder Network, Wikipedia Mathematician network, College Message network, and Email network. Each row is for a different link prediction method, see Table E2 for abbreviations. The precise values are not important here as the important feature is the success or failure to identify two clear groups of low and high scoring node pairs. A successful method should have two separate peaks clearly visible in these plots and only the Katz method shows this on a regular basis.

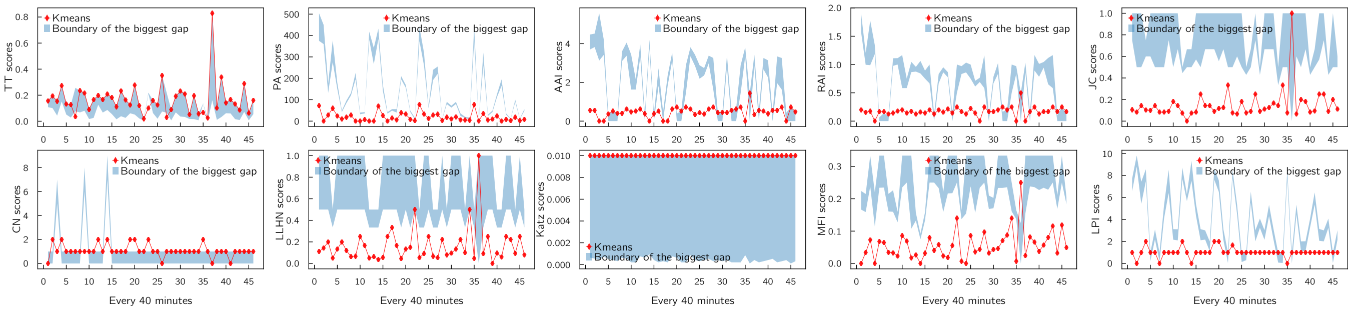

Figure E12: The thresholds evolution plots for Hypertext networks of $dt = 40$ minute. The red line represent the equivalent threshold of Kmeans clustering method which we use to do predictions and the blue filled space represents the boundaries of the biggest gap.

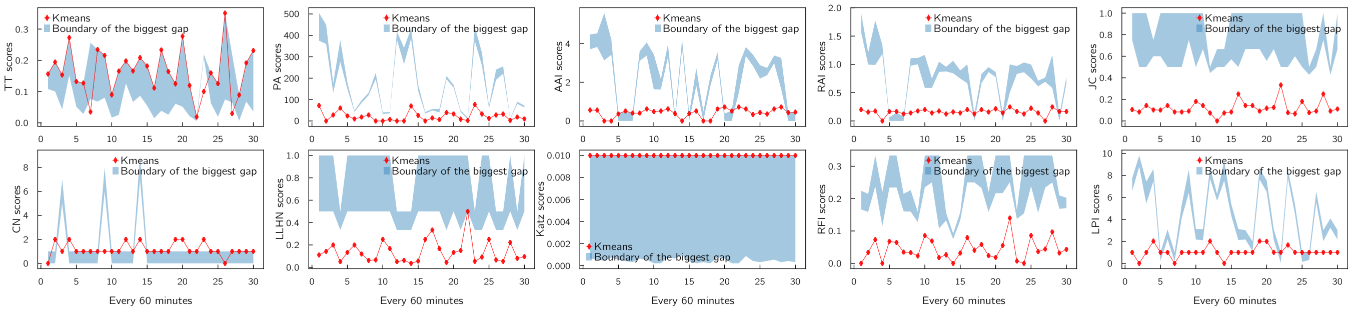

Figure E13: The thresholds evolution plots for Hypertext network of $dt = 60$ minute. The red line represent the equivalent threshold of Kmeans clustering method which we use to do predictions and the blue filled space represents the boundaries of the biggest gap.

F Measures of Success and Baseline Scores

The notation $N_{\alpha\beta\pm}$ is the number of node pairs that

- start in state α (1 if the node pair is connected by an edge, 0 otherwise) in $\mathcal{G}(s)$,
- which change to state β defined in the same way but in terms of the existence of an edge between the same node pair, in snapshot $\mathcal{G}(s+1)$,
- for which the prediction made for that node pair was correct (+) or incorrect (−).

So if the prediction for node pair (i, j) from snapshot s to snapshot $(s+1)$ is $P(i, j)$ then

$$N_{\alpha\beta+} = \sum_{(i,j)} \delta(\alpha, A_{ij}(s))\, \delta(\beta, A_{ij}(s+1))\, \delta(P(i,j), A_{ij}(s+1)) \tag{F17}$$

where $\delta(a, b) = 1$ if $a = b$ otherwise it is zero (the Kronecker delta function). For later convenience we also define

$$N_{\alpha\beta} = N_{\alpha\beta+} + N_{\alpha\beta-} = \sum_{(i,j)} \delta(\alpha, A_{ij}(s))\delta(\beta, A_{ij}(s+1)) \tag{F18}$$

$$N_{\cdot\beta} = N_{0\beta} + N_{1\beta} = \sum_{j} \delta(\beta, A_{ij}(s+1)) \tag{F19}$$

The precision score is the number of times we predict an edge to exist between node pairs in the later snapshot correctly (a true positive) divided by the number of times we predict an edge, correctly (true positive) or incorrectly (false positive)

$$S_{\text{prec}} = \frac{N_{11+} + N_{01+}}{N_{11+} + N_{01+} + N_{11-} + N_{01-}} . \tag{F20}$$

A high precision score means we can trust that edges predicted by the algorithm will exist.

The recall is the fraction of positive identifications which were correct, so

$$S_{\rm rec} \quad = \quad \frac{N_{11+}+N_{01+}}{N_{11+}+N_{01+}+N_{10-}+N_{00-}}\,, \tag{F21}$$

The accuracy is the number of correct predictions (edge or no edge) in the final snapshot over the total number of predictions

$$S_{\rm acc} \quad = \quad \frac{N_{11+}+N_{01+}+N_{10+}+N_{00+}}{N_{11+}+N_{01+}+N_{10+}+N_{00+}+N_{11-}+N_{01-}+N_{10-}+N_{00-}}\,, \tag{F22}$$

Our baseline model is that we predict that an edge will connect a node pair with probability $\rho(s)$ where this is the density of the network in snapshot s, that is the fraction of edge pairs with an edge in snapshot s where for N nodes in the network and ignoring self-loops ($i=j$ excluded)

$$\rho(s) = \frac{\sum_{(i,j)} A_{i,j}}{\sum_{(i,j)} 1} = \frac{2}{N(N-1)} \sum_{(i,j)} A_{i,j}\,. \tag{F23}$$

This baseline model is equivalent to just randomising the edges in snapshot s as a prediction for the next snapshot; it doesn't even preserve the degree of the nodes. This means that in our notation we have that

$$N_{\alpha 1+} = \rho(s) N_{\alpha 1+}\,, \quad N_{\alpha 1-} = (1-\rho(s)) N_{\alpha 1-}\,, \quad N_{\alpha 0+} = (1-\rho(s)) N_{\alpha 1-}\,, \quad N_{\alpha 0-} = \rho(s) N_{\alpha 0-}\,. \tag{F24}$$

The baseline value of precision is

$$S_{\rm prec,base} \quad = \quad \frac{\rho(s) N_{\cdot 1}}{\rho(s) N_{\cdot 1} + (1-\rho(s)) N_{\cdot 1}} \tag{F25}$$

$$= \quad \rho(s)\,. \tag{F26}$$

For recall we find the baseline value is

$$S_{\rm rec,base} \quad = \quad \frac{\rho(s) N_{11} + \rho(s) N_{01+}}{\rho(s) N_{11} + \rho(s) N_{01} + \rho(s) N_{10} + \rho(s) N_{00}} = \frac{N_{\cdot 1}}{N_{\cdot 1} + N_{\cdot 0}} \tag{F27}$$

$$= \quad \rho(s+1)\,. \tag{F28}$$

where $\rho(s+1)$ is the density of edges in the network in snapshot $(s+1)$. Finally accuracy in our baseline model is

$$S_{\rm acc,base} \quad = \quad \frac{\rho(s) N_{\cdot 1} + (1-\rho(s)) N_{\cdot 0}}{\rho(s) N_{\cdot 1} + (1-\rho(s)) N_{\cdot 0} + (1-\rho(s)) N_{\cdot 1} + \rho(s) N_{\cdot 0}} \tag{F29}$$

$$= \quad \rho(s)\rho(s+1) + (1-\rho(s))(1-\rho(s+1))\,, \tag{F30}$$

where for definiteness we have written $\rho = \rho(s)$.

An even more naive model which gives us another reference value would be one in which we simply predict an edge for any given node pair 50% of the time. In our notation, this is equivalent to saying that $N_{\alpha\beta +} = N_{\alpha\beta -}$. In this case the precision (F20) and accuracy (F22) both equal one half while recall (F21) is simply the fraction of connected node pairs in the network, the network density.

G Hypertext Network

For completeness, we give the Hypertext network results equivalent to those shown in Figures 3, 5 and 6 of the main text and in Figures 14 and D10 of the appendix.

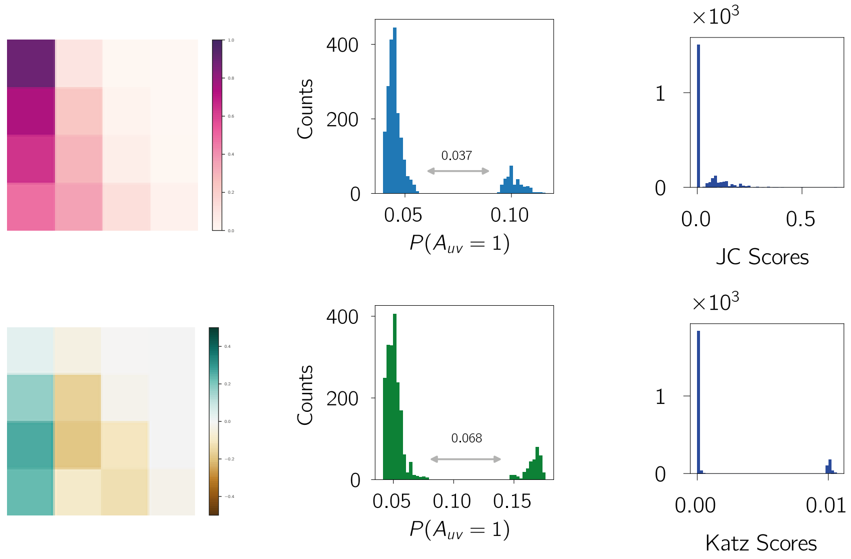

Figure G14: Results for the Hypertext network based using a time window of 60min. Top left we show the transition matrix, bottom left we have a plot showing the entries of $\delta\hat{T}$. The central column shows the node similarity scores in our triplet transition (TT) method for the $\mathcal{M}_4$ (top) and $\mathcal{M}_8$ cases (bottom). The right hand column shows similar results for $\mathcal{M}_4$ when using the Katz (top) and JC (bottom) methods.

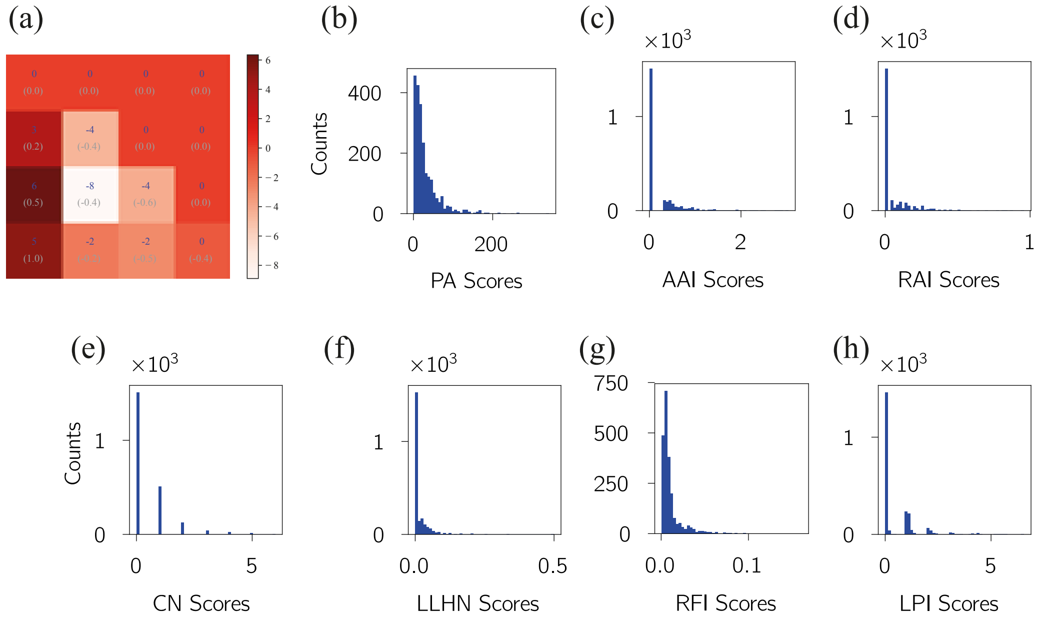

Figure G15: Results for the Hypertext network based using a time window of 60min. Top left (a) we show the Zscore for transition matrix of Figure G14. (b)-(h) show the node similarity scores for other methods.

References

[1] Yao, Q., Evans, T. S., & Christensen, K. How the network properties of shareholders vary with investor type and country. *PloS one* **14** e0220965; 10.1371/journal.pone.0220965 (2019).

[2] Chen,B., Lin,Z. & Evans, T. Analysis of the Wikipedia Network of Mathematicians. Preprint at https://arxiv.org/abs/1902.07622 (2019).

[3] Opsahl, T., & Panzarasa, P. Clustering in weighted networks. *Social networks* **31**, 155–163 (2009).

[4] Panzarasa, P., Opsahl, T., & Carley, K. M. Patterns and dynamics of users' behavior and interaction: Network analysis of an online community. *Journal of the American Society for Information Science and Technology* **60**, 911–932 (2009).

[5] Leskovec, J. & Andrej K. SNAP Datasets: Stanford Large Network Dataset Collection, http://snap.stanford.edu/data (2014).

[6] Leskovec, J., Kleinberg, J., & Faloutsos, C. Graph evolution: Densification and shrinking diameters. *ACM transactions on Knowledge Discovery from Data (TKDD)* **1** (2007).

[7] Yin, R., Benson,A., Leskovec,J., & Gleich,D. Local Higher-order Graph Clustering. In *Proceedings of the 23rd ACM SIGKDD International Conference on Knowledge Discovery and Data Mining*, 555–564 (2017).

[8] Kunegis, J. KONECT-The Koblenz Network Collection. *Proc. Int. Conf. on World Wide Web Companion*, 1343–1350 (2013).

[9] Isella, L.,*et al.* What's in a crowd? Analysis of face-to-face behavioral networks. *Journal of Theoretical Biology* **271**, 166-180 (2011).

[10] Milo, Ron, *et al.* Network motifs: simple building blocks of complex networks. *Science* **298**, 824–827 (2002).

[11] Kovanen, L., Kaski, K., Kertész, J., & Saramäki, J. Temporal motifs reveal homophily, gender-specific patterns, and group talk in call sequences. *Proceedings of the National Academy of Sciences* **110**, 18070–18075 (2013).

[12] Liben-Nowell, D., & Kleinberg, J. The link-prediction problem for social networks. *Journal of the American society for information science and technology* **58**, 1019–1031 (2007).

[13] Lü, L., Jin, C. H., & Zhou, T. Similarity index based on local paths for link prediction of complex networks. *Physical Review E* **80**, 046122 (2009).

[14] Leicht, E. A., Holme, P., & Newman, M. E. Vertex similarity in networks. *Phys. Rev. E* **73**, 026120 (2006).

[15] Zhou, T., Lü, L., & Zhang, Y. C. Predicting missing links via local information. *The European Physical Journal B* **71**, 623–630 (2009).

[16] Chebotarev, P., & Shamis, E. . The matrix-forest theorem and measuring relations in small social groups. Preprint at https://arxiv.org/abs/math/0602070 (2006).

[17] Hagberg ,A., Schult A. & Swart, P., Exploring network structure, dynamics, and function using NetworkX, in *Proceedings of the 7th Python in Science Conference (SciPy2008), Gäel Varoquaux, Travis Vaught, and Jarrod Millman (Eds), (Pasadena, CA USA)*, 11-15(2008).

[18] AbuOda, G., Morales, G. D. F., & Aboulnaga, A. Link prediction via higher-order motif features. In *Joint European Conference on Machine Learning and Knowledge Discovery in Databases*, 412–429 (2019).

[19] Barandela, R., Sánchez, J. S., Garca, V., & Rangel, E., Strategies for learning in class imbalance problems. *Pattern Recognition* **36**, 849–851 (2003).

[20] Salton, G. & McGill, M. J., Introduction to Modern Information Retrieval, *McGrawHill*, 1983.

[21] Sarukkai, R. R. Link prediction and path analysis using markov chains. *Computer Networks* **33**, 377–386 (2000).

[22] Popescul, A., & Ungar, L. H. Statistical relational learning for link prediction. In *IJCAI workshop on learning statistical models from relational data 2003* (2003).

[23] Bilgic, M., Namata, G. M., & Getoor, L. Combining collective classification and link prediction. In *Seventh IEEE International Conference on Data Mining Workshops (ICDMW 2007)* 381–386, IEEE (2007).

[24] Yu, K., Chu, W., Yu, S., Tresp, V., & Xu, Z. Stochastic relational models for discriminative link prediction. In *NIPS* **6**, 1553–1560 (2006).

[25] Clauset, A., Moore, C., & Newman, M. E. Hierarchical structure and the prediction of missing links in networks. *Nature* **453**, 98–101 (2008).
